# Synergistic Control of Kinetochore Protein Levels by Psh1 and Ubr2

**DOI:** 10.1371/journal.pgen.1005855

**Published:** 2016-02-18

**Authors:** Eva Herrero, Peter H. Thorpe

**Affiliations:** The Francis Crick Institute, Mill Hill Laboratory, London, United Kingdom; University of North Carolina, UNITED STATES

## Abstract

The accurate segregation of chromosomes during cell division is achieved by attachment of chromosomes to the mitotic spindle via the kinetochore, a large multi-protein complex that assembles on centromeres. The budding yeast kinetochore comprises more than 60 different proteins. Although the structure and function of many of these proteins has been investigated, we have little understanding of the steady state regulation of kinetochores. The primary model of kinetochore homeostasis suggests that kinetochores assemble hierarchically from the centromeric DNA via the inclusion of a centromere-specific histone into chromatin. We tested this model by trying to perturb kinetochore protein levels by overexpressing an outer kinetochore gene, *MTW1*. This increase in protein failed to change protein recruitment, consistent with the hierarchical assembly model. However, we find that deletion of Psh1, a key ubiquitin ligase that is known to restrict inner kinetochore protein loading, does not increase levels of outer kinetochore proteins, thus breaking the normal kinetochore stoichiometry. This perturbation leads to chromosome segregation defects, which can be partially suppressed by mutation of Ubr2, a second ubiquitin ligase that normally restricts protein levels at the outer kinetochore. Together these data show that Psh1 and Ubr2 synergistically control the amount of proteins at the kinetochore.

## Author Summary

As cells divide, their replicated chromosomes must be correctly allocated to the two nascent daughter cells. This is achieved by the kinetochore, which provides a physical link between the chromosomes and the microtubules that drive their movement. If chromosome separation fails, the resulting cells have an abnormal number of chromosomes. This state is called aneuploidy and is a hallmark of cancer cells. The regulation of the kinetochore is therefore of critical importance in maintaining genome integrity. Since a number of cancer cells have over-active kinetochore genes, it has been proposed that an excess of kinetochore proteins can disrupt the normal assembly or maintenance of kinetochores. We tested this idea in yeast by increasing the amount of a specific kinetochore protein, but found no effect upon the normal loading of kinetochore proteins. Instead, we find that two ubiquitin ligases play a role in maintaining the normal balance of the different kinetochore proteins and that this correlates with correct segregation of the chromosomes.

## Introduction

Accurate chromosome segregation is necessary for the equal distribution of genetic material between daughter cells during cell division and is achieved by kinetochores which link chromosomes to spindle microtubules [1]. Perturbations of kinetochore function result in aneuploidy, i.e. changes in chromosome number, and genome instability [2, 3]. Thus kinetochore regulation is of critical importance in replicating cells. A number of different cancers overexpress kinetochore genes [4, 5] leading to the notion that disrupting kinetochore stoichiometry and regulation may be a driver of aneuploidy and genomic instability.

Budding yeast is a key model to study kinetochore composition and assembly because of its comparatively simple structure; there is only one microtubule attachment per chromosome and per kinetochore [6, 7]. Kinetochores are composed of more than 60 proteins organized into various sub-complexes that are thought to assemble hierarchically initiating at the centromeres [1]. The inner part of the kinetochore mediates centromere binding whereas the outer part mediates microtubule binding. Kinetochore structure and composition is remarkably well conserved from yeast to humans [8].

In budding yeast the position of the centromeres is sequence specific. Cbf1 and the CBF3 complex associate to centromere DNA elements (CDE), CDEI and CDEIII, respectively [9–13]. The CDEII region wraps around the centromeric nucleosome that contains the centromeric histone H3 variant CENP-A (Cse4 in budding yeast) [14–17]. Mif2 (CENP-C) and the COMA complex mediate the association between centromere and outer kinetochore. Mif2 binds to both the Cse4 nucleosome and the outer kinetochore MIND complex [18–20]. The COMA complex proteins Okp1 and Ame1 form a dimer that binds directly to DNA and the MIND complex [20, 21].

The outer kinetochore mediates interactions with microtubules emanating from opposite spindle pole bodies. The yeast homologues of the KNL1/ MIS12/ NDC80 network (KNM) are the essential complexes SPC105, MIND and NDC80, respectively [1]. The MIND complex is composed of two heterodimers: Mtw1-Nnf1, which associates with both Mif2 and the COMA complex, and Dsn1-Nsl1, which associates with the NDC80 complex [21, 22]. Both the NDC80 complex and the yeast-specific DAM-DASH complex, which may play an orthologous function to the human SKA proteins [23], bind to microtubules in a cooperative process [24, 25].

Although the centromeric DNA sequence (CEN) is essential to assemble kinetochores, protein degradation has been shown to be important to control cellular levels of various kinetochore proteins. The E3 ubiquitin ligase Psh1 restricts the localization of Cse4 to centromeres [26]. Psh1 localizes to centromeres throughout the cell cycle, and its destabilizing role is opposed by the Cse4 chaperone Scm3 [27, 28]. Levels of Cse4 are increased in *psh1Δ* cells [26] and these cells have a chromosomal instability phenotype [29]. More recently, the E3 ubiquitin ligase Ubr2 has been shown to control levels of the MIND complex protein Dsn1 [30]. Thus kinetochore assembly may be regulated differently from steady state homeostasis. Surprisingly, yeast kinetochores can assemble in reverse from the microtubule interface back to the inner kinetochore as shown via artificial recruitment of proteins to DNA [31]. In this situation, the conserved yeast centromere is not necessary, although inner kinetochore proteins are required [32]. These data point to a kinetochore with more flexibility in its assembly and stoichiometry than was previously assumed.

Numerous studies in budding yeast have revealed the stoichiometry of the various protein sub-complexes forming the kinetochore [20, 21, 33–37]. It is thought that the kinetochore assembles hierarchically from the centromere [37]. However, little is known about how these sub-complexes assemble to form the kinetochore *in vivo* and how much flexibility exists in kinetochore composition. To investigate this, we tested how increased levels of kinetochore proteins affect kinetochore composition. We used fluorescence microscopy to quantify the levels of proteins at kinetochore foci. We found that Mtw1 levels at the kinetochore correlate with chromosome number and they are not transcriptionally controlled. Moreover, we found that *psh1Δ* mutants, in addition to the elevated Cse4 protein, have increased levels of inner kinetochore proteins but not outer kinetochore proteins. However, the levels of outer kinetochore proteins are increased in the *psh1Δ ubr2Δ* double mutant, in which both Cse4 and Dsn1 are unconstrained. Finally, we found that *ubr2Δ* suppresses *psh1Δ* mitotic and meiotic defects. These findings are consistent with multiple regulatory pathways acting independently on the different kinetochore complexes.

## Results

### Loading of Mtw1 onto kinetochores is not restricted by its gene expression

**Fig 1:**
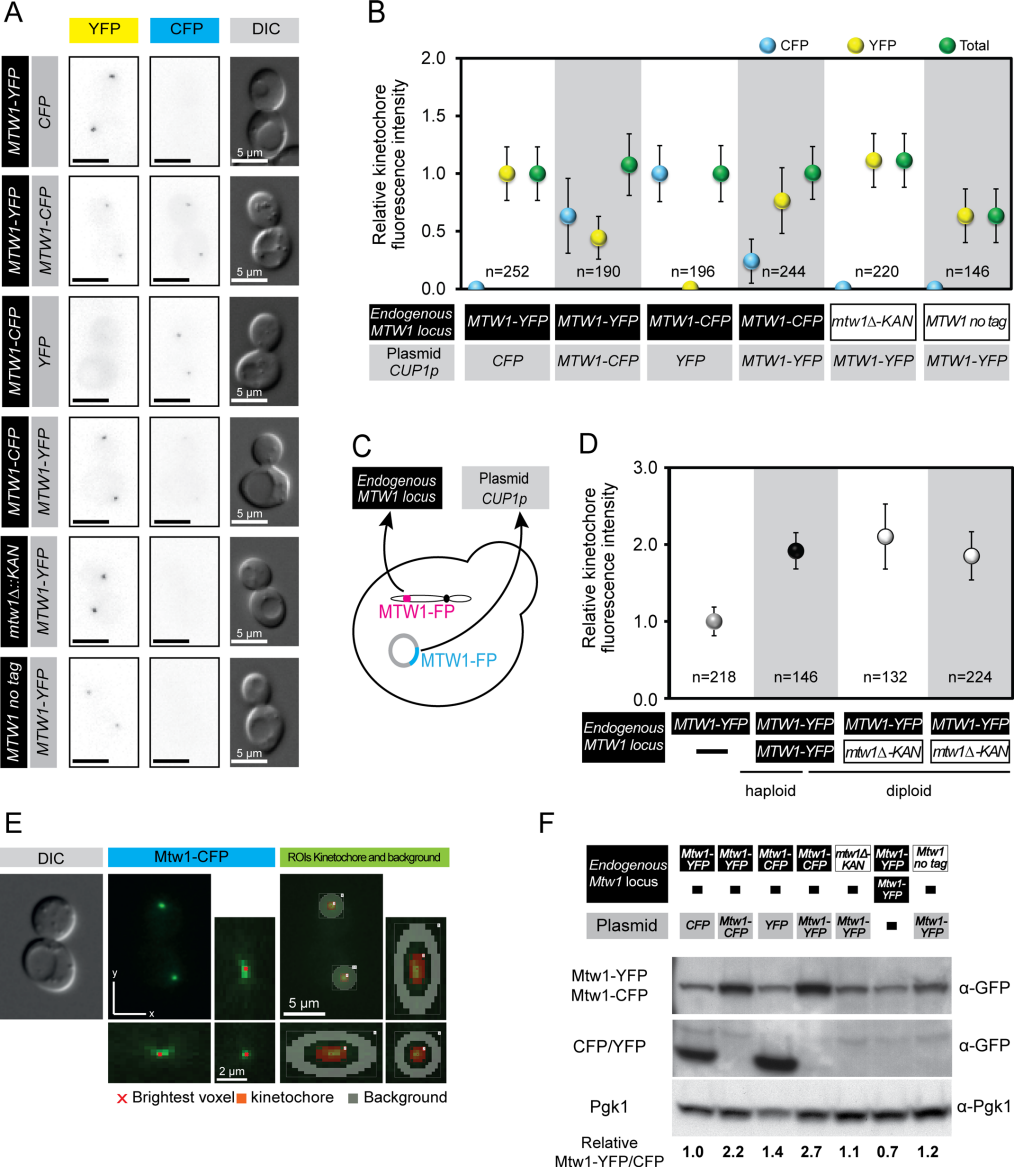
Robust levels of Mtw1 protein at kinetochore foci. (A) Representative images of telophase cells expressing different combinations of endogenous (black box) and ectopic (grey box) Mtw1-YFP and/or Mtw1-CFP tagged proteins (see Fig 1C). (B) Levels of Mtw1 protein at kinetochore foci are plotted relative to the mean intensity of haploid Mtw1-CFP or Mtw1-YFP. Mtw1-YFP (YFP, yellow markers), Mtw1-CFP (CFP, blue markers) and Total (green markers). Error bars indicate standard deviation of the mean. (C) The endogenous *MTW1* locus was tagged with the gene encoding either YFP or CFP, or it was deleted (*mtw1Δ*). Ectopic Mtw1-YFP or Mtw1-CFP was expressed from a CEN plasmid under the control of the *CUP1* promoter (no copper was added to the media). (D) Mtw1 levels at the kinetochore correspond with chromosome number. Levels of Mtw1 at kinetochore foci are plotted relative to mean intensity in haploid Mtw1-YFP cells. Error bars indicate standard deviation of the mean. (E) Strategy used for automatic identification of kinetochore and background regions and quantification of fluorescence at kinetochore foci using Volocity software. The region of interest (ROI) quantified for the kinetochore is highlighted in red and the background ROI highlighted in grey. (F) Total Mtw1 protein increases by ectopic expression of *MTW1*. Western blot of total cell extracts. Quantification of cellular levels of Mtw1-YFP/-CFP relative to Pgk1 is shown below. An extended blot is shown in S2A Fig.

To investigate whether we could perturb kinetochore homeostasis by overexpression of kinetochore genes, we chose to study *MTW1*. Mtw1 forms part of the essential MIND complex [21, 38] and the levels of one of these proteins, Dsn1, is controlled via phosphorylation status and subsequent ubiquitylation by the E3 ligase, Ubr2 [30]. We used an ectopically-expressed plasmid-encoded version of Mtw1 to elevate the levels of Mtw1 within the cell and assessed the recruitment of Mtw1 to kinetochores by fluorescence imaging. The plasmid is a single copy *CEN* plasmid and its *MTW1* gene is driven by a constitutively-active copper promoter (*CUP1*) [39]. We used differential fluorescence tagging of endogenously-encoded and plasmid-encoded Mtw1 to differentiate between and quantitate the proteins loaded into kinetochores (Fig 1A, 1B and 1C). The *MTW1* plasmid produced significant ectopic expression as judged by loading of plasmid-encoded Mtw1 at the kinetochore (Fig 1A). We quantified the levels of fluorescence at kinetochores using Volocity image analysis software. In brief, the mean fluorescence within a 3-dimensional spherical region around each kinetochore was assessed and a background region around each kinetochore was also measured by dilating each kinetochore selection (Fig 1E). Each background measurement was subtracted from each kinetochore measurement to produce a relative value representing the levels of fluorescence signal from the kinetochore. When we expressed an ectopic *MTW1-CFP* gene in cells containing *MTW1-YFP* at the endogenous locus, we found that the resulting fluorescence at kinetochores was approximately 50% of the haploid CFP signal and 50% of the haploid YFP signal (Fig 1B). This is consistent with an approximately equal contribution of the two proteins to the kinetochore, but not consistent with an elevation of Mtw1 loading at the kinetochore. To determine whether one fluorescent tag is preferred over the other, we then performed the same analysis but with the tags reversed i.e. ectopic *MTW1-YFP* and endogenous *MTW1-CFP*. In this case the levels of the plasmid encoded Mtw1-YFP at the kinetochore are somewhat higher than the CFP signal, although both still contribute to the kinetochore signal (Fig 1B). Again, no increase in total kinetochore fluorescence was measured. We also examined the effect of deleting the endogenous *MTW1* gene in cells containing an *MTW1-YFP* plasmid. The level of YFP fluorescence in this stain is the same as an endogenously-encoded *MTW1-YFP* strain, (Fig 1B). Finally, we transformed the *MTW1-YFP* plasmid into an untagged strain. We find that the Mtw1-YFP level of fluorescence is equivalent to the strain with both endogenously and ectopically-encoded Mtw1, approximately 50% (Fig 1B). We also assessed whether changes in the background levels of fluorescence in the cells over-expressing kinetochore proteins were increased, resulting in an artificially low kinetochore signal. However, we find that changes to background fluorescence do not mask an effect of *MTW1* expression on kinetochore protein levels (S1A and S1B Fig). Thus, these quantitative data support the notion that the fluorescently tagged proteins compete for inclusion into the kinetochore and that the total levels of kinetochore Mtw1 remain constant. There are two likely reasons for this homeostasis of Mtw1 at the kinetochore. First, an uncharacterised negative feedback mechanism could limit transcription, translation or protein stability of the endogenous Mtw1, thus maintaining a steady state level of Mtw1 protein within the cell. Second, the loading of Mtw1 onto the kinetochores is limiting, such that there is a strong affinity to load Mtw1 as part of the MIND complex but once the protein reaches a threshold level (perhaps through stoichiometric interaction with other kinetochore components), no more Mtw1 is loaded. To discriminate between these two ideas we used western blotting to assess the total cellular levels of Mtw1. We find that the ectopic expression of *MTW1* causes an increase in the levels of Mtw1 protein in the cell (Fig 1F). Thus, we exclude the possibility that total Mtw1 protein levels are tightly regulated by translation or protein stability.

Our results are also consistent with the notion of hierarchical assembly of the kinetochore building up from inner kinetochore components such as Cse4. To test this notion we compared the loading of Mtw1 in diploid strains with *MTW1-YFP* at either one or two of the endogenous *MTW1* alleles. We find that diploid kinetochore Mtw1 levels are approximately double that of haploids and heterozygous *mtw1Δ/MTW1-YFP* strains compensate by loading equivalent Mtw1 as diploid strains (Fig 1D). We note here that these heterozygous *mtw1Δ/MTW1-YFP* strains are haplo-sufficient in that they do not show sensitivity to microtubule poison drug benomyl (S2B Fig). We also confirmed that overexpression of *MTW1* does not render cells sensitive to benomyl (S2C Fig), nor does it affect cell cycle progression (S3A Fig), plasmid loss (S3B Fig), or chromosome segregation (S3C and S3D Fig). We also checked whether *MTW1* overexpression resulted in changes to the levels of other kinetochore proteins and consistent with the levels of Mtw1, we find no change in Dsn1 or Ndc80 (S3E and S3F Fig). In order to test more generally the effects of high levels of kinetochore proteins, we expressed various inner and outer kinetochore proteins from a *CEN* plasmid under the control of a *CUP1* promoter. Only *NDC10* overexpression showed a reduced growth in the presence of benomyl (S4 Fig) We then tested whether Mtw1 kinetochore levels were affected by the deletion of genes encoding several inner kinetochore components: the DNA-binding protein Cbf1, the Monopolin complex components Mam1 and Csm1, and the COMA complex component Ctf19. We found no change in Mtw1 levels in any of these mutants (S5A and S5B Fig), consistent with Mtw1 loading hierarchically based upon the number of centromeres present in the cell.

### Elevated Cse4 levels increase loading of inner kinetochore proteins

The hierarchical loading model is consistent with the hypothesis that the loading of inner kinetochore proteins is critical for determining kinetochore stoichiometry as a whole. To test this idea we decided to attempt to manipulate the levels of an inner kinetochore protein to test whether the MIND complex is regulated in parallel.

**Fig 2:**
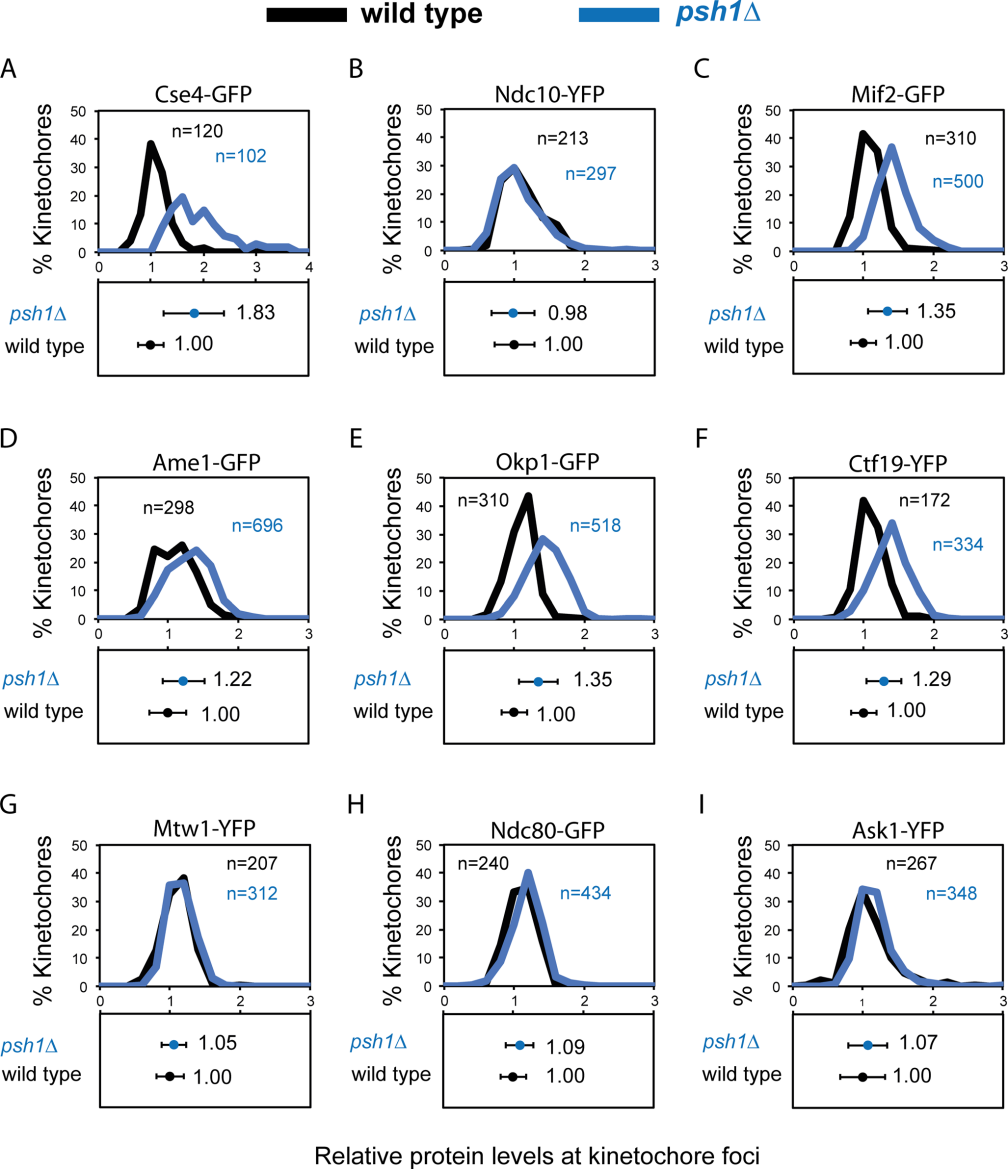
Increased levels of Cse4 does not affect all kinetochore protein complexes. Quantitation of protein levels at kinetochore foci in wild type (black) and *psh1Δ* (blue) cells. Fluorescence intensity levels are normalised relative to wild-type mean intensity ± standard deviation. Top panels and bottom panels display the distribution of intensities and the mean intensity ± standard deviation, respectively. (A) Cse4-GFP wild type 1.00±0.25, *psh1Δ* 1.83±0.57***. (B) Ndc10-YFP wild type 1.00±0.29, *psh1Δ* 0.98±0.31. (C) Mif2-GFP wild type 1.00±0.18, *psh1Δ* 1.35±0.25***. (D) Ame1-GFP wild type 1.00±0.26, *psh1Δ* 1.22±0.30***. (E) Okp1-GFP wild type 1.00±0.18, *psh1Δ* 1.35±0.28***. (F) Ctf19-YFP wild type 1.00±0.18, *psh1Δ* 1.29±0.25***. (G) Mtw1-YFP wild type 1.00±0.19, *psh1Δ* 1.05±0.18. (H) Ndc80-GFP wild type 1.00±0.19, *psh1Δ* 1.07±0.22. (I) Ask1-YFP wild type 1.00±0.32, *psh1Δ* 1.07±0.28. ***p-value <0.0001 is a *t* test comparing relative intensity levels of wild-type and *psh1Δ* cells.

The levels of the inner kinetochore protein Cse4 are controlled in part by degradation via an ubiquitylation-dependent degradation pathway. Psh1 was identified as the E3 ubiquitin ligase responsible for restricting Cse4 levels at the kinetochore [26, 27]. In a *psh1Δ* strain Cse4 levels are elevated and furthermore overexpression of the *CSE4* is lethal in *psh1Δ* cells, consistent with a failure to constrain Cse4 loading [26, 27]. We used the same fluorescence quantitation method described above to compare endogenous kinetochore protein levels of wild-type cells with those of *psh1Δ* cells. Consistent with previous studies we find that *psh1Δ* cells have elevated levels of Cse4 at kinetochore foci, although with considerable heterogeneity between cells (Fig 2A). We found no change in the protein levels of the inner kinetochore protein Ndc10 (Fig 2B). In addition, we find that Mif2, the ortholog of human CENP-C, (Fig 2C) and members of the Ctf19/COMA complex are also elevated in the *psh1Δ* (Fig 2D, 2E and 2F). However, contrary to our expectation Mtw1 kinetochore levels are unchanged in a *psh1Δ* strain compared with wild type (Fig 2G). We therefore examined whether other outer-kinetochore complexes are affected by deletion of *PSH1*. Like Mtw1, the kinetochore levels of Ndc80 and Ask1 (a member of the decameric DAM1/DASH complex) are both unaffected in *psh1Δ* cells (Fig 2H and 2I). These data show that although Cse4 levels may influence the inner kinetochore, the protein levels of the entire kinetochore are not affected. This result shows that for the fluorescence focus that is widely considered to represent the structural kinetochore the stoichiometry is not fixed.

**Fig 3:**
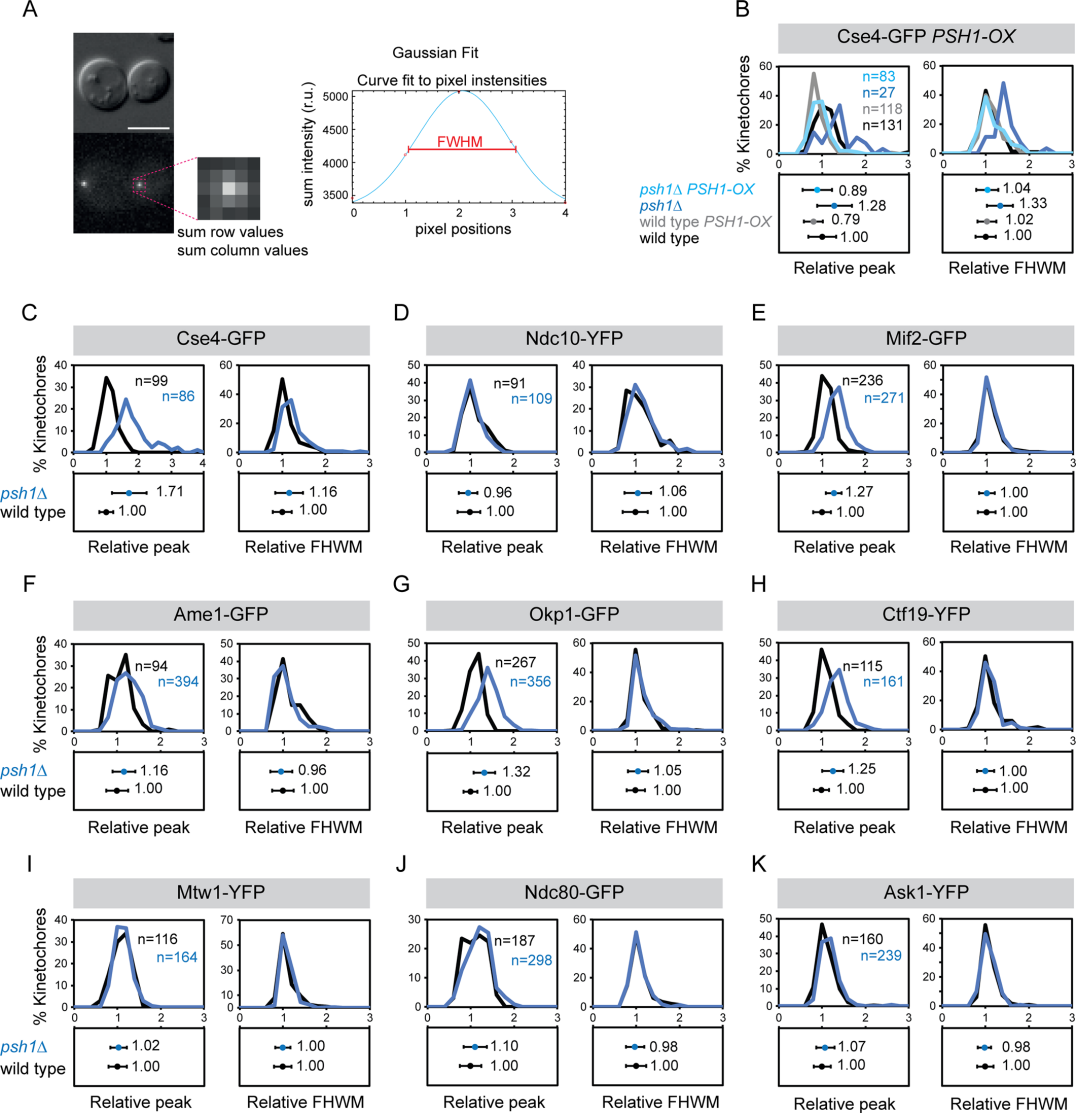
Changes in the size of kinetochore foci in *psh1Δ* mutant. (A) Strategy to fit Gaussian distribution to kinetochore foci. (B-K) Quantitation of size of kinetochore foci in wild type (black) and *psh1Δ* (blue) cells. The images used for Fig 2 quantitation were re-analysed to calculate peak height and FHWM shown in panels C-K. Fluorescence peak height values and full width at half maximum (FHWM) values are normalised relative to wild-type mean intensity ± standard deviation. Top panels and bottom panels display the distribution of intensities and the mean intensity ± standard deviation, respectively. (B) Cse4-GFP PSH1-OX. Peak height: wild type 1.00±0.32, wild type *PSH1-OX* 0.79±0.22, *psh1Δ* 1.28±0.41, psh1*Δ PSH1-OX* 0.89±0.34. FWHM: wild type 1.00±0.23, wild type *PSH1-OX* 1.02±0.22, *psh1Δ* 1.33±0.30, psh1*Δ PSH1-OX* 1.04±0.26. (C) Cse4-GFP. Peak height: wild type 1.00±0.22, *psh1Δ* 1.71±0.53***. FHWM: wild type 1.00±0.23, *psh1Δ* 1.16±0.32***. (D) Ndc10-YFP. Peak height: wild type 1.00±0.24, *psh1Δ* 0.96±0.21. FHWM: wild type 1.00±0.29, *psh1Δ* 1.06±0.31. (E) Mif2-GFP. Peak height: wild type 1.00±0.17, *psh1Δ* 1.27±0.26***. FHWM: wild type 1.00±0.21, *psh1Δ* 1.00±0.18. (F) Ame1-GFP. Peak height: wild type 1.00±0.25, *psh1Δ* 1.16±0.26***. FHWM: wild type 1.00±0.25, *psh1Δ* 0.96±0.26. (G) Okp1-GFP. Peak height: wild type 1.00±0.16, *psh1Δ* 1.32±0.25***. FHWM: wild type 1.00±0.21, *psh1Δ* 1.05±0.24. (H) Ctf19-YFP. Peak height: wild type 1.00±0.17, *psh1Δ* 1.25±0.25***. FHWM: wild type 1.00±0.26, *psh1Δ* 0.96±0.2. (I) Mtw1-YFP. Peak height: wild type 1.00±0.21, *psh1Δ* 1.02±0.19. FHWM: wild type 1.00±0.20, *psh1Δ* 1.00±0.18. (J) Ndc80-GFP. Peak height: wild type 1.00±0.25, *psh1Δ* 1.10±0.27. FHWM: wild type 1.00±0.23, *psh1Δ* 0.98±0.21. (K) Ask1-YFP. Peak height: wild type 1.00±0.2, *psh1Δ* 1.07±0.22. FHWM: wild type 1.00±0.19, *psh1Δ* 0.98±0.15.

One possible reason for the non-stoichiometric increase in kinetochore protein levels in *psh1Δ* cells is that the increased Cse4, Ctf19 etc. are not part of the canonical kinetochore structure, but rather represent a pericentromeric ‘cloud’ of protein. There is precedent for this from fluorescence studies of Cse4 [40, 41]. We therefore re-analysed our images to evaluate the size each of the fluorescence foci. The rationale is that pericentric protein recruitment will result in a larger area of fluorescence, which can be measured by fitting a Gaussian distribution to the kinetochore foci (Fig 3A). We find that *psh1Δ* Cse4 foci are considerably larger than WT, consistent with the notion of a cloud of pericentric Cse4 and this is rescued by overexpressing *PSH1* (Fig 3B and 3C). However, the other kinetochore proteins had *psh1Δ* foci comparable in size to WT cells (Fig 3C–3K). We cannot say for sure that protein that is located in a comparably-sized focus is part of a structural complex, it is possible that for certain proteins the kinetochore can accommodate additional proteins within the confines of the WT diffraction limited region.

### Psh1 and Ubr2 work together to control kinetochore proteins levels

**Fig 4:**
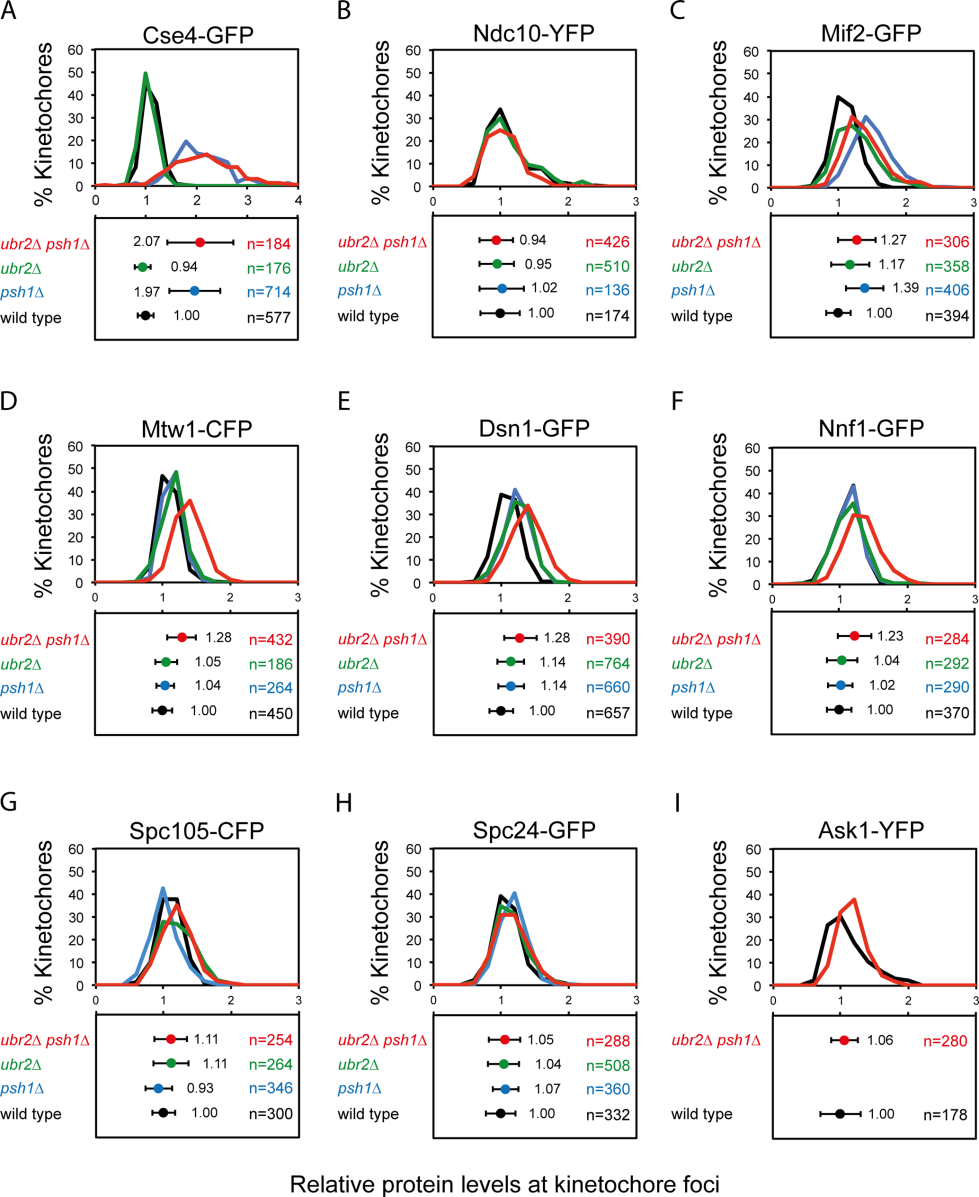
Psh1 and Ubr2 work together to control kinetochore protein levels. Quantitation of protein levels at kinetochore foci in wild type (black), *psh1Δ* (blue), *ubr2Δ* (green) and *psh1Δ ubr2Δ* (red). Fluorescence intensity levels are normalised relative to wild-type mean intensity. Top panels and bottom panels display the distribution of intensities and the mean intensity ± standard deviation, respectively. (A) Cse4-GFP wild type 1.00±0.16, *psh1Δ* 1.97±0.50***, *ubr2Δ* 0.94±0.16, *psh1Δ ubr2Δ* 2.07±0.66***. (B) Ndc10-YFP wild type 1.00±0.29, *psh1Δ* 1.02±0.25, *ubr2Δ* 0.95±0.33, *psh1Δ ubr2Δ* 0.94±0.25. (C) Mif2-GFP wild type 1.00±0.18, *psh1Δ* 1.39±0.28***, *ubr2Δ* 1.17±0.29, *psh1Δ ubr2Δ* 1.27±0.28***. (D) Mtw1-CFP wild type 1.00±0.14, *psh1Δ* 1.04±0.13, *ubr2Δ* 1.05±0.16, *psh1Δ ubr2Δ* 1.28±0.21***. (E) Dsn1-GFP wild type 1.00±0.18, *psh1Δ* 1.14±0.19, *ubr2Δ* 1.14±0.20, *psh1Δ ubr2Δ* 1.28±0.24***. (F) Nnf1-GFP wild type 1.00±0.18, *psh1Δ* 1.02±0.18, *ubr2Δ* 1.04±0.23, *psh1Δ ubr2Δ* 1.23±0.25***.(G) Spc105-GFP wild type 1.00±0.17, *psh1Δ* 0.93±0.20, *ubr2Δ* 1.11±0.26, *psh1Δ ubr2Δ* 1.11±0.24. (H) Spc24-GFP wild type 1.00±0.21, *psh1Δ* 1.07±0.19, *ubr2Δ* 1.04±0.23, *psh1Δ ubr2Δ* 1.05±0.23. (I) Ask1-YFP wild type 1.00±0.3, *psh1Δ ubr2Δ* 1.06±0.20. ***p-value <0.0001 is a *t* test comparing relative mean intensity of wild type and mutant.

**Fig 5:**
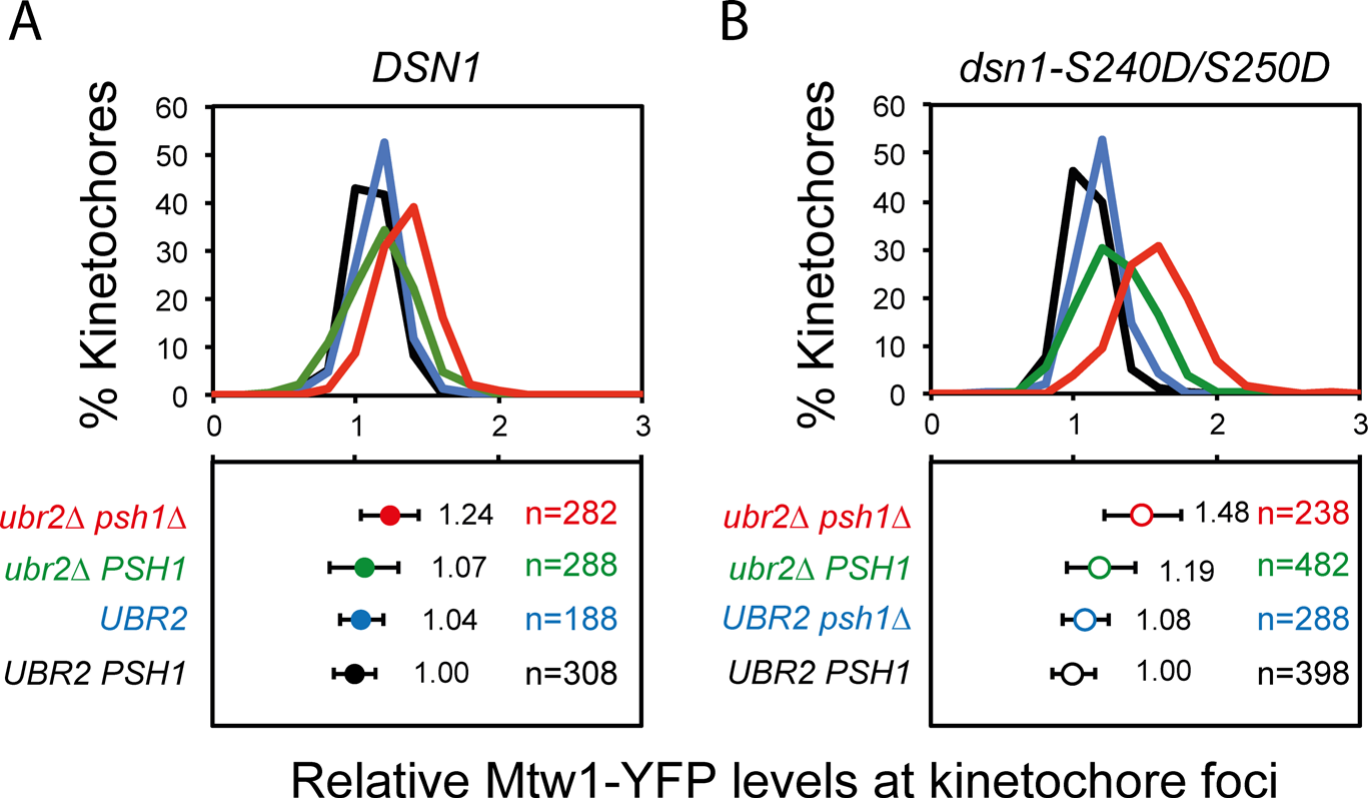
*ubr2Δ* and *dsn1-S240D/S250D* phospho-mimic mutants do not function epistatically. Quantitation of Mtw1-YFP levels at kinetochore foci in wild type (black), *psh1Δ* (blue), *ubr2Δ* (green) and *psh1Δ ubr2Δ* (red): Fluorescence intensity levels are normalised relative to wild-type mean intensity. Top panels and bottom panels display the distribution of intensities and the mean intensity ± standard deviation, respectively. (A) Strains contain wild type *DSN1*: *PSH1 UBR2* 1.00±0.14, *psh1Δ UBR2* 1.04±0.18, *PSH1 ubr2Δ* 1.07±0.24, *psh1Δ ubr2Δ* 1.24±0.2***. (B) Strains contain the *dsn1-S240D/S250D* allele: *PSH1 UBR2* 1.00±0.15, *psh1Δ UBR2* 1.08±0.16, *PSH1 ubr2Δ* 1.19±0.24, *psh1Δ ubr2Δ* 1.48±0.27***. ***p-value <0.0001 is a *t* test, comparing the levels of Mtw1-YFP in *PSH1 UBR2* cells with *psh1Δ ubr2Δ* double mutant cells (both for wild-type *DSN1* and for the *dsn1-S240D/S250D* strain). For *psh1Δ ubr2Δ* cells the *dsn1-S240D/S250D* mutant had higher levels of Mtw1-YFP than the DSN1 (1.24±0.2 *vs*. 1.48±0.27 *p*-value <0.0001).

We next asked whether the effect of Psh1 upon kinetochore protein levels would function in synergy with the Mub1/Ubr2 ubiquitylation pathway. The MIND complex member Dsn1 is ubiquitylated by the E3 ubiquitin ligase Ubr2 [30]. Dsn1 contains two AuroraB (Ipl1) phosphorylation sites (serines 240 and 250) and versions of Dsn1 that cannot be phosphorylated at these residues are ubiquitylated and degraded [30, 42]. Such a mechanism may restrict the levels of MIND proteins even in the presence of excess inner kinetochore proteins. Since *psh1Δ*, *ubr2Δ* and the double mutant cells are all viable we were able to assess their relative contribution to the kinetochore focus fluorescence levels. We find that *UBR2* deletion has no effect upon inner kinetochore protein levels of Cse4 or Ndc10. Cse4 levels are elevated by *PSH1* deletion, but not further affected by the additional deletion of *UBR2* (Fig 4A). Also addition of *ubr2Δ* mutation did not further increase the size of Cse4-GFP foci (S6A Fig). Ndc10 is unaffected by either of these mutants (Fig 4B). Mif2 is elevated in a *psh1Δ* mutant, but unaffected by further deletion of *UBR2* (Fig 4C). The MIND complex shows little change in either of the single mutants but both Mtw1 and Dsn1 are modestly elevated in the double *psh1Δ ubr2Δ* strain (Fig 4D and 4E). The size of Mif2 and Dsn1 foci was unaffected in the *ubr2Δ* and in the double *psh1Δ ubr2Δ* cells (S6B and S6C Fig). Another MIND complex protein Nnf1 is also elevated in *psh1Δ ubr2Δ* cells (Fig 4F). Other outer kinetochore proteins Spc105, Spc24, from NDC80 complex, and Ask1 were unaffected by either of these mutants (Fig 4G, 4H and 4I). The degradation of Dsn1 is controlled by phosphorylation/ dephosphorylation of serines 240 and 250. The double *dsn1-S240A*,*S250A* mutant is inviable, but can be rescued by either its overexpression or by deleting *UBR2* [30]. We reasoned that if increased Dsn1 was responsible for the MIND phenotype, this should be epistatic with a *dsn1-S240D*,*S250D* mutant, which would be hyper-stable. However, we find that the elevated levels of Mtw1 in a *psh1Δ ubr2Δ* mutant are increased further when the two Dsn1 serines are changed to aspartic acid (Fig 5A and 5B). Furthermore, we examined cellular levels of both Mtw1 and Dsn1 in *psh1Δ*, *ubr2Δ* and the *psh1Δ ubr2Δ* mutants and find that these are comparable with wild-type cells (S6D and S6E Fig) These data suggest that Ubr2 plays additional, potentially indirect, roles in regulating the levels of kinetochore components in addition to its function on dephosphorylated Dsn1 or that there are other mechanisms to remove dephosphorylated Dsn1 from kinetochores. These data also strengthen our observation that the stoichiometry of the various kinetochore sub-complexes is not fixed in these mutants.

### *ubr2Δ* supresses *psh1Δ* chromosome instability phenotypes

**Fig 6:**
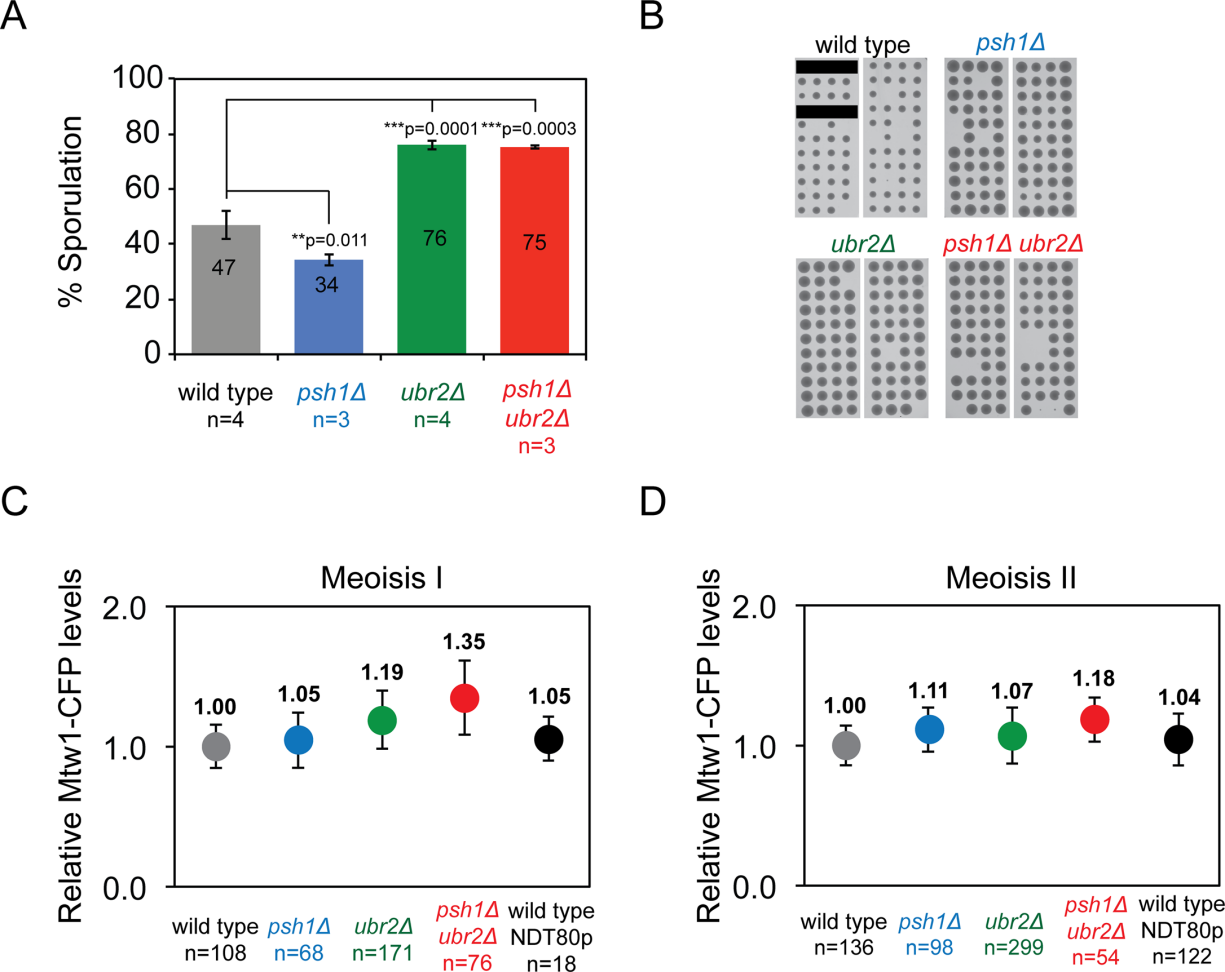
Meiotic phenotypes of *psh1Δ* and *ubr2Δ* ubiquitin ligase mutants. (A) The percentage of cells that sporulated after three days in sporulation media at 23°C. Error bars indicate standard deviation of the mean. *p*-values in the graph correspond to *t* test comparing mean % sporulation. Wild type 47%±5.27, *psh1Δ* 34%±2.02, *ubr2Δ* 76%±1.65, and *psh1Δ ubr2Δ* 75%±0.77. (B) Spore germination of tetrads dissected from wild-type and mutant diploid strains show equivalent spore viability. Black bars indicate that no tetrad was placed in the line. Wild type (73/80), *psh1Δ* (84/88), *ubr2Δ* (85/88), and *psh1Δ ubr2Δ* (79/88). (C-D), Quantitation of Mtw1-CFP levels at kinetochore foci in Meiosis I (C) and Meiosis II (D). Fluorescence intensity levels are plotted relative to wild-type mean intensity and error bars indicate standard deviation of the mean. Meiosis I: wild type 1±0.15, *psh1Δ* 1.05±0.20, *ubr2Δ* 1.19±0.21, *psh1Δ ubr2Δ* 1.35±0.26***. Meiosis II: wild type 1±0.14, *psh1Δ* 1.11±0.16, *ubr2Δ* 1.07±0.2, *psh1Δ ubr2Δ* 1.18±0.16***. ***p-value <0.0001 is a *t* test comparing relative mean intensity of wild-type and *psh1Δ ubr2Δ* mutant cells.

Although these ubiquitin ligase mutants affect kinetochore protein levels, they are all viable and the cells appear to grow normally [26, 30]. Since there is considerable interest in the possibility that altered kinetochore protein levels would lead to kinetochore dysfunction and the resulting aneuploidy [4, 5, 43], we asked whether the *psh1Δ* and *ubr2Δ* mutants affected the mitotic or meiotic phenotype of yeast. We did not find strong defects in cell cycle progression, although S-phase was slightly faster in *ubr2Δ* and *psh1Δ ubr2Δ* mutants (S7 Fig). It has previously been reported that *ubr2Δ* mutants have an enhanced sporulation phenotype [44]. Consistent with this we found that the sporulation of homozygous *ubr2Δ* mutants is enhanced compared with wild-type diploids (Fig 6A). Addition of the *psh1Δ* mutant did not modify this phenotype. In all cases spore viability was similar (Fig 6B). We tested whether the increase in Mtw1 kinetochore levels in *psh1Δ ubr2Δ* mitotic cells (Fig 3D) was recapitulated in meiosis. Diploid cells were induced to sporulate and arrested in pachytene, prior to the two meiotic divisions by depletion of the Ndt80 transcription factor. Then, meiosis I was triggered by induction of *NDT80* expression from the GAL1-10 promoter [45] (see Materials and Methods for details). We found elevated Mtw1 kinetochore levels in *psh1Δ ubr2Δ* in meiosis I, and to a lesser extent in meiosis II (Fig 6C and 6D).

**Fig 7:**
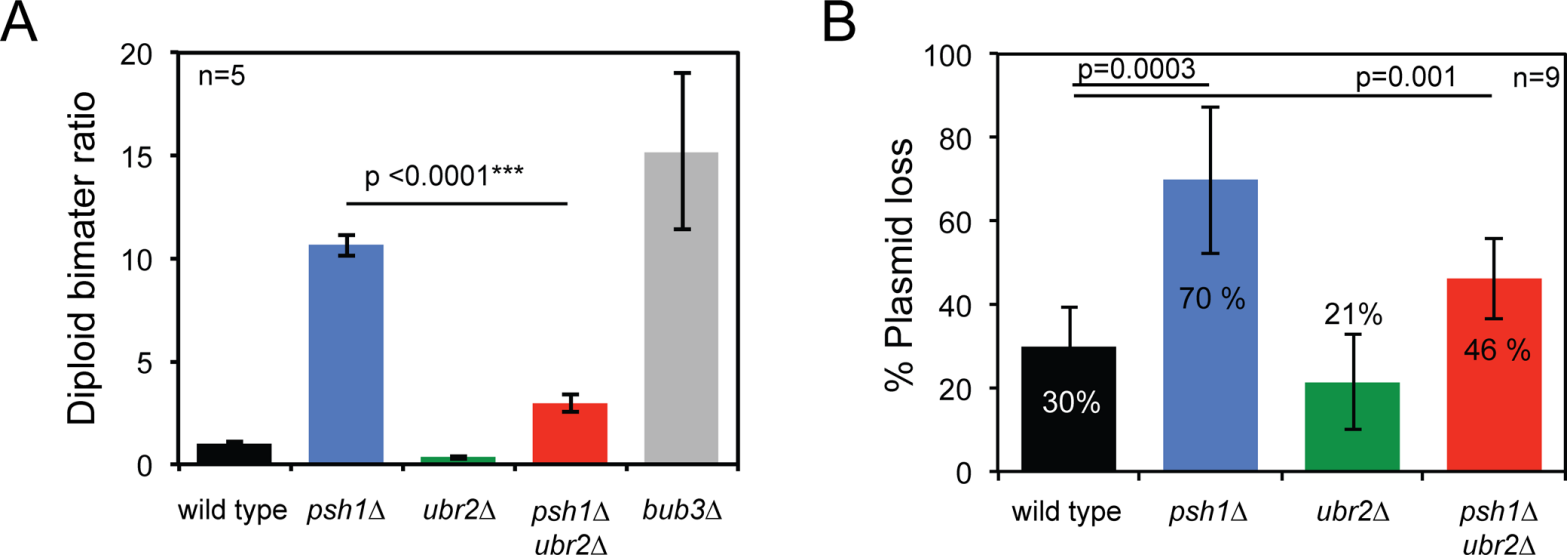
*ubr2Δ* supresses *psh1Δ* chromosome instability phenotype. (A) Diploid- bi-mater ratio. Median bimater ratio was calculated from 5 independent colonies from each genotype. Median bimater ratio ± standard error of the mean: wild type 1±0.07, *psh1Δ* 10.7±0.54, *ubr2Δ* 0.4±0.09, *psh1Δ ubr2Δ* 3±0.41 and *bub3Δ* 15.2±3.8. *p*-values in the graph correspond to *t* test comparing bimater ratios. (B) CEN plasmid loss assay. Median percentage of plasmid loss was calculated from 9 independent colonies from each genotype. Median plasmid loss ± standard deviation: wild type 30±9, *psh1Δ* 70±17, *ubr2Δ* 21±11, *psh1Δ ubr2Δ* 46±. *p*-values in the graph correspond to t-test comparing plasmid loss percentages.

**Fig 8:**
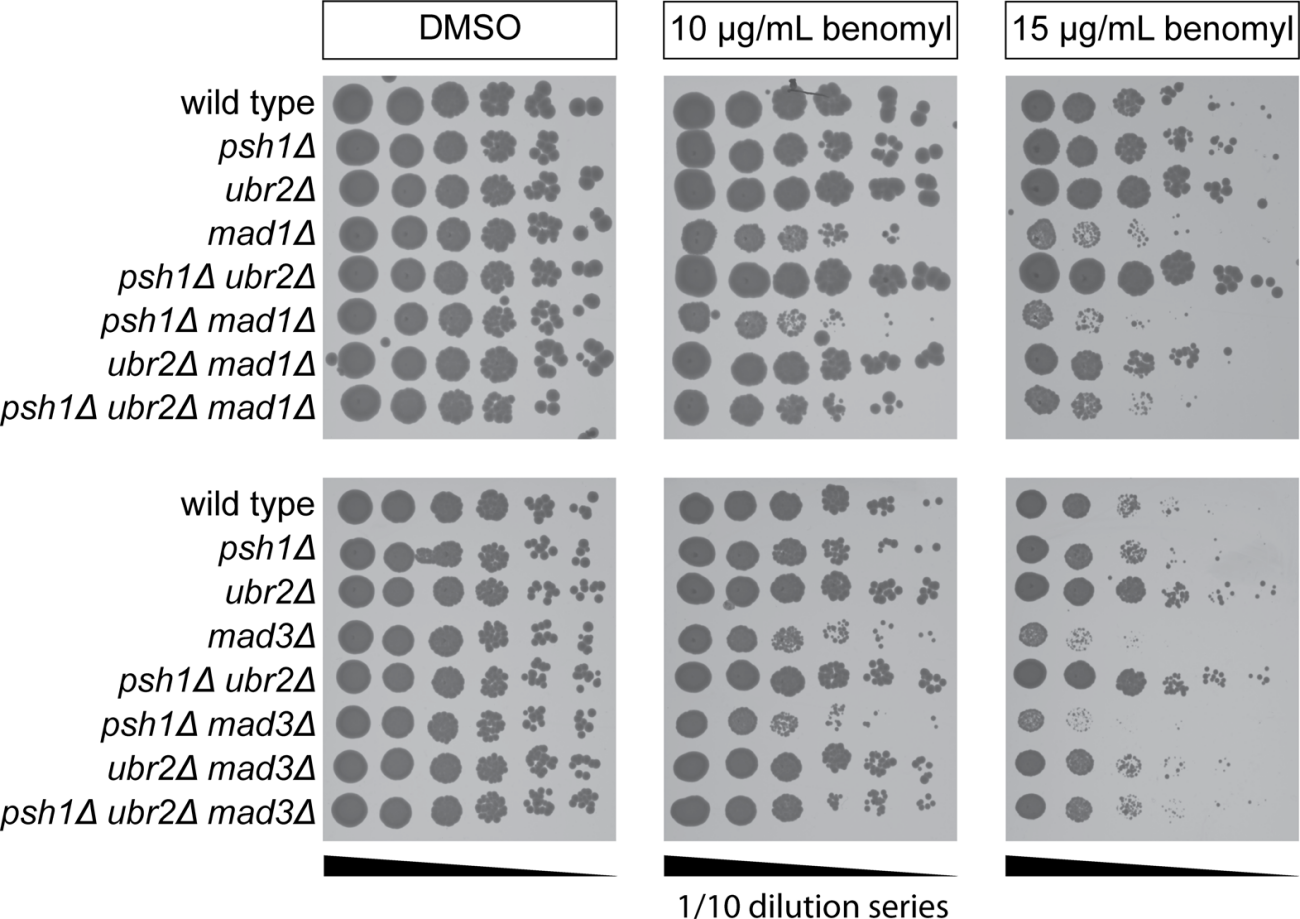
Genetic interaction of *psh1Δ* and *ubr2Δ* with the spindle assembly checkpoint. Serial dilutions of cells were spotted into YPD plates with and without benomyl and then grown for 2 days at 30°C prior to imaging.

As Psh1 is known to have a role in maintaining chromosome stability [29], we used an assay for homozygosity of chromosome III [2, 3, 29] to analyse the rate of chromosomal instability (CIN) in diploids cells, and we also tested the rate of loss of a CEN plasmid. Consistent with previous reports, we find that *psh1Δ* cells show elevated rates of both chromosome III loss (Fig 7A) and *CEN* plasmid loss (Fig 7B), whereas *ubr2Δ* cells are unaffected. Surprisingly, we found that the addition of *ubr2Δ* to a *psh1Δ* mutant leads to a reduction of these CIN phenotypes (Fig 7A and 7B). To investigate the effect of the ubiquitin ligases Psh1 and Ubr2 on checkpoint function, we assessed the synthetic effects of combining mutations in these genes with those of checkpoint genes. We deleted the *MAD1* gene, which encodes a protein required for the activation of Mad2 [46] and also *MAD3*, which encodes a key member of the mitotic checkpoint complex [47]. These mutants were combined with *psh1Δ*, *ubr2Δ* or the double mutant. The resulting strains were all viable (Fig 8), so to test their checkpoint proficiency we grew them in the microtubule poison benomyl. We found that deletion of *psh1Δ* decreases the ability of both *mad1Δ* and *mad3Δ* to grow in the presences of benomyl (Fig 8). Moreover, deletion of *ubr2Δ* partially rescued the ability of *mad1Δ* and *mad3Δ* to grow on benomyl. Finally, we also found that *ubr2Δ* partially rescues the benomyl sensitivity of *mad1Δ psh1*Δ and *mad3Δ psh1Δ* double mutants (Fig 8). We then tested if increased Dsn1 levels could explain the rescue of *ubr2Δ*. However, we found that *DSN1* over-expression from a *CUP1* promoter did not rescue benomyl sensitivity (S8 Fig)

## Discussion

A number of studies have shown correlation between the overexpression of kinetochore genes and tumorigenic status [4, 5, 43]. These observations raise the possibility that increased levels of kinetochore proteins result in aberrant kinetochore function, which then leads to chromosomal instability. We wished to test the idea that overexpression of kinetochore genes would affect kinetochore protein loading. We overexpressed the kinetochore gene, *MTW1* that encodes a core member of the outer kinetochore MIND complex. The MIND complex plays an essential role in linking the inner kinetochore and the outer kinetochore [48, 49]. Using quantitative fluorescence imaging we find that although overexpression of *MTW1* does lead to increased Mtw1 protein in the cell, the loading of Mtw1 onto the kinetochores is unaffected (Fig 1). Our data supports the idea that kinetochores are assembled hierarchically from the inner kinetochore, likely directed by Cse4 inclusion into centromeric nucleosomes [37]. Similarly, Aravamudhan and colleagues found that the levels of Cse4 at the kinetochore did not change after increasing total Cse4 cellular levels in budding yeast [50]. The effects of kinetochore gene overexpression may be subtle and/or different in mammalian cells, however, our data do not support the idea that kinetochore gene overexpression would, *a priori*, lead to a kinetochore defect (Figs 1, S2–S4). On the contrary, our data also support the idea that the kinetochore focus represents the structural assembly of kinetochore proteins loaded onto centromeres [37, 51] and that kinetochore protein levels scale with centromere number (Fig 1) [52]. However, recent work using synthetic kinetochores has demonstrated that a functional kinetochore can assemble backwards from the microtubule associated DAM1/DASH complex [31, 32]. Recruitment of outer kinetochore proteins to a non-centromere sequence is sufficient to generate an artificial kinetochore that no longer requires a specific *CEN* sequence but does require inner kinetochore proteins. These observations challenge the hierarchical assembly model, albeit in an artificially tethered system and suggest that the kinetochore structure may be more adaptable than previously imagined.

In an effort to perturb the kinetochore structure we examined kinetochores in mutants of two ubiquitin ligases that are known to affect the degradation of kinetochore proteins, Psh1 and Ubr2. The Psh1 ubiquitin ligase regulates the levels of Cse4 protein at the kinetochore focus [26, 27]. We confirmed that the levels of Cse4 are increased in *psh1Δ* cells, and additionally found that the levels of inner kinetochore proteins Mif2, Okp1, Ame1 and Ctf19 also increase (Fig 2). The increase in kinetochore-loaded Cse4 was higher than the other inner kinetochore proteins, suggesting that some of the excess Cse4 is not able to recruit these additional proteins and maybe part of a local ‘cloud’ of Cse4 adjacent to the kinetochore [40] or that it is in a form that is unable to recruit the other components. Consistent with the former notion, we find that the increased Cse4 in a *psh1Δ* mutant is spread over a larger area, although this is not true for all kinetochore proteins that are elevated in *psh1Δ* cells (Fig 3). This may explain why a large increase in Cse4 levels results in only a modest increase in, for example, members of the COMA complex. Surprisingly, we found that outer kinetochore protein levels are unaffected in *psh1Δ* cells (Fig 2). These data support the idea that in these mutants the stoichiometry of the kinetochore is flexible. We found that mutating both *PSH1* and *UBR2* is sufficient to modestly increase the levels of members of the MIND complex (Fig 4). In budding yeast, if we assume two Cse4 molecules per centromere, there are about 6–7 MIND complexes per kinetochore in anaphase [7, 53]. In the *psh1Δ ubr2Δ* double mutants, the ~ 30% increase of Mtw1 and Dsn1 would correspond to ~2 additional MIND complexes per kinetochore. It is unlikely that the chromosome instability phenotype found in *psh1Δ and psh1Δ ubr2Δ* (Fig 7) accounts for the difference in kinetochore protein levels (Fig 2 and Fig 4). If these mutant cells would have a higher number of chromosomes (due to their CIN phenotype), we would expect all kinetochore components to be similarly increased. Instead, we find no change in Ndc10 protein levels in the absence of Psh1, Ubr2 or both (Fig 2 and Fig 4), and we also did not find an increase in the outer kinetochore proteins in *psh1Δ* cells. It is possible that the additional proteins at the kinetochore focus in *psh1Δ* and *psh1Δ ubr2Δ* are not part of the structural kinetochore assembly. However, the magnitude of the increase of Mtw1 and Dsn1 in the *psh1Δ ubr2Δ* double mutant (Fig 4) is similar to the increase in Mif2 and COMA complex proteins in the *psh1Δ* mutant (Fig 2). This suggests that the amount of MIND complex binding to the kinetochore is still limited by the amount of inner kinetochore components, consistent with a hierarchical kinetochore assembly. The double *psh1Δ ubr2Δ* mutant does suppress some characteristics of the *psh1Δ* phenotype; including meiotic sporulation defects (Fig 6) and mitotic genome instability (Fig 7). It is possible that partially restoring the stoichiometry between inner and outer kinetochore proteins contributes to this phenotypic suppression. However, it is important to note that there is no evidence that the increased Cse4 levels at the kinetochore in *psh1Δ* cells cause their CIN phenotype. Collectively our data show that inclusion of kinetochore proteins into the kinetochore focus is flexible in mutant backgrounds. Furthermore, that the genomic instability of *psh1Δ* cells, which may result from increased Cse4 loading, is suppressed by second mutation, *ubr2Δ*, that also increases the levels of MIND complex members.

In *psh1Δ* cells, Cse4 is increased at kinetochore foci (Fig 2) and also deposited ectopically in non-centromeric regions [26, 27]. Both kinetochore and non-kinetochore ectopic pools of Cse4 could contribute to *psh1Δ* chromosomal instability phenotype [29] (Fig 6). The negative interaction of *psh1Δ* with spindle assembly checkpoint components *mad1Δ* and *mad3Δ* in the presence of microtubule poison (Fig 8) suggests a decreased kinetochore function in *psh1Δ*. Surprisingly, *ubr2Δ* partially rescued benomyl sensitivity of both *mad1Δ* and *mad3Δ* also in combination with *psh1Δ* (Fig 8). This *ubr2Δ* suppressor effect was not recapitulated by *DNS1* overexpression (S8 Fig), suggesting an additional role of Ubr2. It is possible that the upregulation of other Ubr2/Mub1 complex targets, such as Rpn4 [54] and Sml1 [55], contribute to the suppression of mitotic and meiotic phenotypes of *ubr2Δ*.

Ubr2 has been previously shown to reduce Dsn1 protein stability by ubiquitylation [30], but the impact of Ubr2 in kinetochore composition was not known. Ipl1 phosphorylation on Dsn1 promotes the interactions of the MIND complex with the inner kinetochore proteins [42]. However, the presence of *dsn1-S240D/S250D* did not increase Mtw1 kinetochore levels in wild type or *psh1Δ* cells, but only in *psh1Δ ubr2Δ* double mutant and slightly in *ubr2Δ* (Fig 5). Our data suggest an important role of Ubr2 on limiting outer kinetochore loading by restricting MIND complex availability (Figs 4 and 5). From our data, we cannot be sure whether the changes in kinetochore protein levels are a direct result of changes in ubiquitylation status of kinetochore proteins, the effects may be indirect. We note that the artificial recruitment of Ubr2 and Mub1 to kinetochores does not cause a growth defect [56]. Our data also show that Ubr2 is upstream of Ipl1 in the regulation of outer kinetochore assembly (Fig 5).

Regardless of the mechanism of action of Psh1 and Ubr2, the flexibility of kinetochore stoichiometry may have some functional significance. Kinetochore components are remarkably well conserved from *S*. *cerevisiae* to *H*. *sapiens* although the centromeres to which they bind are highly divergent both in length and sequence. It is hard to imagine that an inflexible kinetochore structure would be sufficient to support the rapid evolution that is typically seen for centromere sequences [57, 58]. Our data in yeast show that overexpression of the kinetochore gene *MTW1* is not sufficient to disrupt kinetochore function, however this may not be true for all kinetochore genes or in nascent tumor cells. This is further supported by the observation that overexpression of *CSE4* is not lethal without further perturbations to the kinetochore [26, 27, 59].

## Materials and Methods

### Yeast strains and plasmid construction

Yeast strains used in this study are either W303 or S288C background, as indicated in S1 Table. For plasmid construction (see S2 Table), the *SPC42-RFP* sequence containing 200 bp of the *SPC42* promoter was cloned into pX29 plasmid (*CEN6*, *LEU2*, *CUP1* promoter). Then, *YFP* (pHT5), *CFP* (pHT222), *MTW1-YFP* (pHT15) or *MTW1-CFP* (pHT223) were cloned downstream of the *CUP1* promoter by gap repair. A sequence encoding four alanine residues was used as a linker between *MTW1* and the fluorescent tags, and between *SPC42* and *RFP*. Plasmids were transformed into appropriate strains by lithium acetate transformation and continuously selected in synthetic media lacking leucine.

*MTW1*, *PSH1* and *UBR2* genes were disrupted by transforming with PCR products containing either *MX6-KAN* or *MX6-NAT* selection cassettes flanked with ~250 bp of sequences upstream and downstream the corresponding coding regions. Gene deletions were confirmed by PCR. Since *MTW1* is an essential gene, it was disrupted in a haploid strain harbouring *CUP1-pMTW1-YFP*::*LEU2* plasmid (pHT15). Transformants were selected in synthetic media lacking leucine and containing G418 and confirmed by PCR. Diploid strain *MTW1-YFP/MTW1-CFP* (PT11) was transformed using *mtw1Δ*::*KANMX* PCR to obtain heterozygous diploids MTW1-YFP/*mtw1Δ*::*KANMX* (PT69 and PT70). Loss of CFP or YFP kinetochore foci was tested by fluorescence microscopy and insertion of the *KANMX* cassette at one of the *MTW1* locus was confirmed by PCR.

### Yeast growth conditions

For microscopy and western blot analysis cells were grown in synthetic complete (SC) or lacking leucine SC–LEU media supplemented with 100mg/ml of adenine (+ADE, 100 mg/mL). Yeast strains were grown overnight at 23°C. Cultures were diluted in fresh media to ≈ OD_600_ 0.3 and grown for 3 hours before imaging or protein extraction.

### Fluorescence microscopy

Cells from log-phase cultures were mounted on microscope slides with 0.7% LMP agarose in SC +ADE or SC-LEU +ADE, and covered with 0.17 mm glass coverslips. Our microscope system uses a Zeiss AxioImager Z2 microscope, 63X Plan Apo, 1.4NA, oil immersion objective and a Hamamatsu CCD ORCAII camera (2X2 binning and maximum analog gain). The resulting pixel size was 0.205 μm. Excitation light was provided by LED Colibri system (excitation band-pass filter): CFP 445 nm (445/25), YFP 505 nm (510/15), GFP 470 nm (474/28) and RFP 590 nm (585/35). Emission band-pass filters were as follows: CFP 47HE (480/40), YFP 46HE (535/30), GFP 38HE (525/50), and RFP 63HE (629/62). Exposure times were optimized for each fluorescent protein and ranged from 100 to 250ms. Z stacks consisted of 17 vertically separated slices with 0.4 μm spacing. The theoretical dynamic range of our system is ~3000 levels of brightness, however, in practice this will be somewhat lower.

### Fluorescence quantitation

A custom-made protocol in Volocity software was used to quantify fluorescence intensity at kinetochore foci. The protocol finds the brightest spots in the image. Spots within 3 pixels from x,y,z edges of the image were removed from the analysis. A 3D box was drawn concentric to the brightest pixels (1.36 μm^3^). The background region was 2 pixels separated from the kinetochore box (23.51 μm^3^). Average intensity of the background was subtracted from average kinetochore intensity to obtain the final fluorescence value. Finally, fluorescence values were normalized to the average of wild type or control populations. For quantitation, only post-anaphase kinetochores of dividing cells were selected.

### Gaussian distribution fitting

To measure the size of individual kinetochore foci we fit two Gaussian distributions to each kinetochore. A five pixel square box was selected for each kinetochore and a local background subtracted. The pixel values in each column and each row were summed and for both the rows and columns and then we used ImageJ’s fitDoFit function to fit a Gaussian curve to the values, separately both the rows and columns (Fig 3A). The two values for the full width at half maximum (FWHM), vertical and horizontal Gaussian fits, were averaged to give a mean FWHM measurement for each focus. The mean FWHM measurements for each experiment were normalized relative to the level in WT cells.

### Western blot analysis

Cell were harvested by centrifugation and resuspended in 1.5X Laemmli buffer with protease inhibitors (Roche) and transferred to a fresh tube containing 0.5 mm glass beads. Cells were disrupted with a cell homogenizer. Cells extracts were harvested into a fresh tube and boiled for 5 minutes. Cells debris was pelleted and 20 μL of the protein extracts were loaded in a 12% acrylamide gel (Biorad). Proteins were transferred into a PVDF blotting membrane (GE Healthcare Amersham). The western blot was performed with monoclonal anti-GFP antibody (Roche), anti-PGK1 (Invitrogen), goat anti-mouse HRP antibody (Abcam), and ECL kit (GE Healthcare Amersham).

### Benomyl sensitivity assay

Yeast strains were grown o/n at 30°C in YPD or selective media. Cultures were adjusted to OD_600_ = 1, serially diluted and spotted into YPD or selective media plates with 0.2% DMSO and 10–15 μg/ml benomyl. For testing effects of overexpression increasing concentrations of CuS0_4_ were added to the media as indicated. Plates were incubated for 2 days at 30°C before images were captured.

### Sporulation efficiency

Diploid strains were grown in YPD at 23°C for 24 hours. Then, cultures were diluted 100X in YEPA media and grown at 23°C until OD_600_ reached 0.6 (2X10^7^ cells/ml). Cultures were washed once with water, resuspended in SPO media and incubated at 23°C for 3 days. Four independent cultures were tested for each genotype. To test spore viability, 22 tetrads per genotype were dissected in YPD and grown for 2 days at 30°C.

### Meiotic synchronization

Diploid strains were grown in YPD for 24 hours at 30°C. Cultures were diluted to OD_600_ 0.3 in YPA (1% yeast extract, 2% Bacto-peptone, 1% potassium acetate) and grown for 12–15 h at 30°C. Cells were then resuspended in sporulation media (1% potassium acetate pH7) at 23°C for 12 hours. Finally, 1μM β-estradiol (Sigma) was added to induce *NDT80* expression. Cells were imaged every hour to follow meiotic divisions.

### Cell cycle analysis

*MAT***a** strains lacking the Bar1 protein were used to facilitate α-factor G1 synchronization. Strains were grown overnight at 30°C, diluted to OD_600_ = 0.3 and grown for 1 hour. The asynchronous sample was collected at this time, then α-factor was added and cells were incubated for additional for 2.5 hours. G1 arrest was confirmed by the presence of the characteristic ‘shmoo’ morphology. Cells were washed twice with water and resuspended in YPD with Pronase E. Samples were taken every 30 minutes until 180 minutes. Cells were prepared for flow cytometry as in [60]. Briefly, cells were fixed overnight in 70% ethanol at 4°C, washed once with water, resuspended in RNAase solution and incubated at 37°C for 2 hours. Cells were then washed once with water and resuspended in protease solution for 30 minutes. For FACS analysis, cells were resuspended in 1μM SYTOX solution (Invitrogen). Cell cycle profiles were generated in a BD Canto Flow cytometer using the GFP filter. G1, S and G2/M populations were calculated using FCS Express (De Novo Software). For S3A Fig, cell cycle progression was scored by fluorescence microscopy. Cells containing a single Mtw1-YFP (kinetochore) and Spc42-RFP (spindle pole body, SPB) foci and without bud were scored as G1 cells. Budding cells with a single kinetochore and SPB were scored as S phase. Cells with one kinetochore and two SPB or two kinetochores and two SPBs were scored as G2/M (Metaphase to Telophase).

### Diploid bimater assay

Diploid *his3*^*-*^*/HIS1* strains were streaked on fresh YPD plates and grown for 2 days at 30°C. Five colonies of each strain were resuspended in YPD. 3x10^6^ cells were mixed with 3x10^7^ cells of log-phase cultures of haploid mating tester strains (*HIS3/his1*^*-*^). Cells were concentrated by gentle centrifugation and incubated overnight at 23°C. The next day these cells were plated on synthetic dropout plates and incubated for 3 days at 30°C to select for *HIS*^+^ mating products. For each colony, mating products originating from both mating type *MAT***a** and *MATα* tester strains were summed. For each strain, the median number of colonies from the 5 colonies was calculated.

### Twin spot assay

Strains with a tetracycline operator array, inserted at the URA3 locus of chromosome V and a tetracycline repressor linked to mRFP, were grown overnight in synthetic media at 23°C. The day after the culture was diluted and further grown until log phase. Cells were imaged as explain above. In each image, cells showing aberrant chromosome segregation were identified as containing two TetR-mFRP foci in G1 or S-M

### Plasmid loss assay

Strains were transformed with a *CEN* plasmid with a selectable marker and grown for two days. 9 colonies were grown overnight in YPD and then plated in either YPD or selective media. The percentage of plasmid loss was calculated by subtracting the amount of cells growing in the selective media to the number of cells growing in YPD. The data is presented as the median of percentage plasmid loss of 9 colonies.

## Supporting Information

S1 Fig Cellular background does not affect Mtw1-YFP quantitation. (A-B) Quantitation of Mtw1-YFP kinetochore and background fluorescence intensity. Top panels and bottom panels display the distribution of intensities and the mean intensity ± standard deviation. Fluorescence intensity levels are normalised relative to the mean intensity of the endogenously tagged Mtw1-YFP strain (black line and circle). Using a background correction region further from kinetochore did not change quantitation of Mtw1-YFP. Strains ectopically expressing Mtw1-YFP have higher background when *MTW1* is also expressed from endogenous locus (green and blue lines and circles).
(TIF)

S2 Fig Ectopic expression of *MTW1* increases Mtw1 cellular levels. (A) Total Mtw1 protein increases in haploid and diploid strains from Fig 1. This is an expanded version of Fig 1F, showing a western blot of total cell extracts from both haploid and diploid cells. Quantification of cellular levels of Mtw1-YFP/-CFP relative to Pgk1 is shown below. (B) Diploid *MTW1-YFP*/*mtw1Δ* are haplo-sufficient. Serial dilutions of cells grown in YPD were spotted onto YPD plates containing benomyl diluted in DMSO. Cells were grown for 2 days at 30°C. (C) *MTW1* over-expression does not affect growth or benomyl sensitivity.
(TIF)

S3 Fig Ectopic expression of *MTW1* does not affect cell cycle progression, chromosome stability and segregation, and kinetochore protein levels. (A) Cell cycle progression is not altered in cells expressing ectopic *MTW1*. (B) The median proportion of cells losing a copy of a CEN plasmid after overnight growth without selection was not significantly different between cells containing an empty plasmid (control) and those containing *MTW1 (MTW1-OX)* (n = 9, error bars show standard deviation of the mean). (C) A tetracycline operator array, inserted at the *URA3* locus of chromosome V, is marked with a tetracycline repressor linked to mRFP. Both normal and aberrant segregation of the chromosome V marker were seen in cells containing an empty plasmid (left panels) and *MTW1* (right panels). Cell outlines are shown in the RFP image as dashed lines, arrowheads highlight aberrant segregation, the scale bar is 5μm. (D) The proportion of cells showing aberrant chromosome V segregation was not significantly different between cells containing an empty plasmid (control) and those containing *MTW1 (MTW1-OX)* (error bars show 95% binomial confidence intervals). (E-F) Quantitation of Dsn1-GFP (E) and Ndc80-GFP (F) kinetochore levels in control (black), low *MTW1-OX* (light blue) and high *MTW1-OX* (dark blue). Fluorescence intensity levels are normalised relative to control mean intensity. Left panel and right panels display the mean intensity ± standard deviation and the distribution of intensities, respectively. Ectopic *MTW1* was expressed from a CUP1p. No additional copper was added to the low *MTW1-OX* cells. 100 μM CuSO_4_ was added to the high *MTW1-OX cells* for 3 hours before imaging.
(TIF)

S4 Fig Ectopic expression of kinetochore proteins do not generally affect growth or benomyl sensitivity. Serial dilutions of cells were spotted into synthetic media lacking leucine to select for plasmid, with several concentrations of benomyl and CuSO_4._ Cells spots were grown for 2 days at 30°C prior to imaging.
(TIF)

S5 Fig Mtw1 kinetochore levels are not affected in various kinetochore mutants. (A-B) Quantitation of Mtw1 kinetochore levels in *ctf19Δ*, *csm1Δ*, *cbf1Δ*, *mam1Δ and cnn1Δ* mutants. Fluorescence intensity levels are normalised relative to wild-type mean intensity. Top panels and bottom panels display the distribution of intensities and the mean intensity ± standard deviation, respectively.
(TIF)

S6 Fig Size of kinetochore foci and MIND protein levels in *psh1Δ* ubr2*Δ* mutant. (A-C) Quantitation of size of kinetochore foci in wild type (black) *psh1Δ* (blue), *ubr2Δ* (green) and *psh1Δ ubr2Δ* (red) cells. Fluorescence peak height values and full width at half maximum (FHWM) values are normalised relative to wild-type mean intensity ± standard deviation. Top panels and bottom panels display the distribution of intensities and the mean intensity ± standard deviation, respectively. (A) Cse4-GFP. Peak height: wild type 1.00±0.15, *psh1Δ* 1.72±0.37***, *ubr2Δ* 0.96±0.14, *psh1Δ ubr2Δ* 1.91±0.48***. FHWM: wild type 1.00±0.26, *psh1Δ* 1.16±0.31***, *ubr2Δ* 0.98±0.25, *psh1Δ ubr2Δ* 1.17±0.28***. (B) Mif2-GFP. Peak height: wild type 1.00±0.24, *psh1Δ* 1.36±0.43***, *ubr2Δ* 1.16±0.28, *psh1Δ ubr2Δ* 1.28±0.30***. FHWM: wild type 1.00±0.25, *psh1Δ* 1.02±0.23, *ubr2Δ* 1.05±0.24, *psh1Δ ubr2Δ* 1.05±0.21. (C) Dsn1-GFP. Peak height: wild type 1.00±0.19, *psh1Δ* 1.12±0.21, *ubr2Δ* 1.06±0.23, *psh1Δ ubr2Δ* 1.18±0.28***. FHWM: wild type 1.00±0.21, *psh1Δ* 0.97±0.17, *ubr2Δ* 1.03±0.24, *psh1Δ ubr2Δ* 1.05±0.23 (D-E) Total Mtw1 and Dsn1 protein do not change in *psh1Δ*, *ubr2Δ* and *psh1Δ ubr2Δ* cells. Western blot of total cell extracts. Quantification of cellular levels of Mtw1-YFP/-CFP relative to Pgk1 is shown below.
(TIF)

S7 Fig Cell cycle progression is not affected in *psh1Δ* and *ubr2Δ* ubiquitin ligase mutants. (A) Cell cycle profiles of wild type, *phs1Δ*, *ubr2Δ*, and *psh1Δ ubr2Δ*. *MAT***a** *bar1Δ* cells were synchronized in G1 with alpha-factor, and then released (Time 0). (B) Changes in the G1, S and G2/M populations during the course of the experiment.
(TIF)

S8 Fig *Dsn1-OX* does not rescue *psh1Δ genetic* interactions with the spindle assembly checkpoint. Serial dilutions of cells were spotted into YPD plates containing NAT to select for *CUP1*p *DSN1*-OX plasmid, with several concentrations of benomyl and CuSO_4._ Cells spots were grown for 2 days at 30°C prior to imaging.
(TIF)

S1 Table List of strains used in this study. (XLSX)

S2 Table List of plasmids used in this study. (XLSX)
