# Supplementary figures and images for: Synergistic Control of Kinetochore Protein Levels by Psh1 and Ubr2
Source: PLoS Genet. 2016 Feb 18;12(2):e1005855. doi: 10.1371/journal.pgen.1005855 (PMC4758618; doi:10.1371/journal.pgen.1005855)

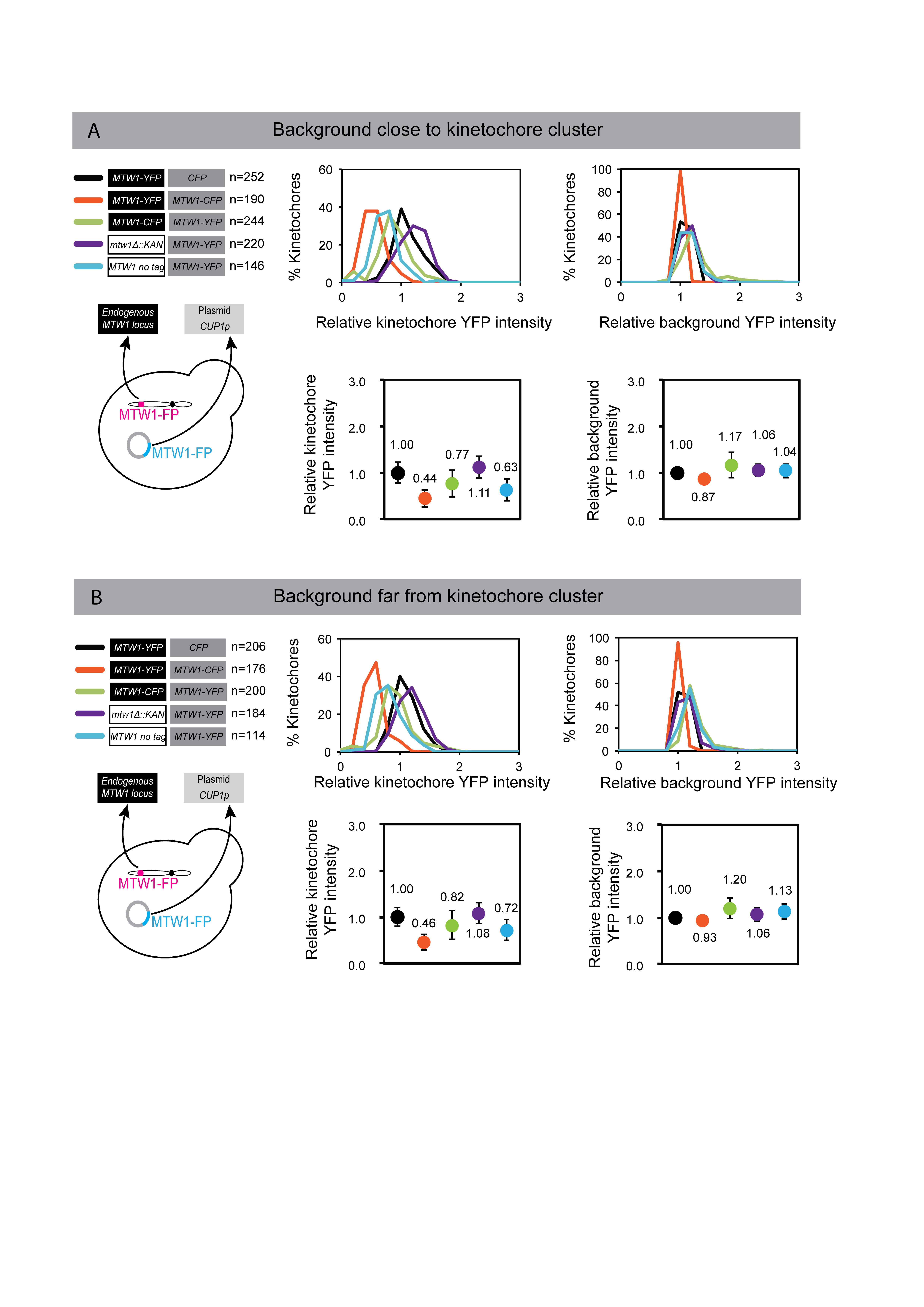

Supplement: S1 Fig — (A-B) Quantitation of Mtw1-YFP kinetochore and background fluorescence intensity. Top panels and bottom panels display the distribution of intensities and the mean intensity ± standard deviation. Fluorescence intensity levels are normalised relative to the mean intensity of the endogenously tagged Mtw1-YFP strain (black line and circle). Using a background correction region further from kinetochore did not change quantitation of Mtw1-YFP. Strains ectopically expressing Mtw1-YFP have higher background when MTW1 is also expressed from endogenous locus (green and blue lines and circles). (TIF) [file pgen.1005855.s001.tif]

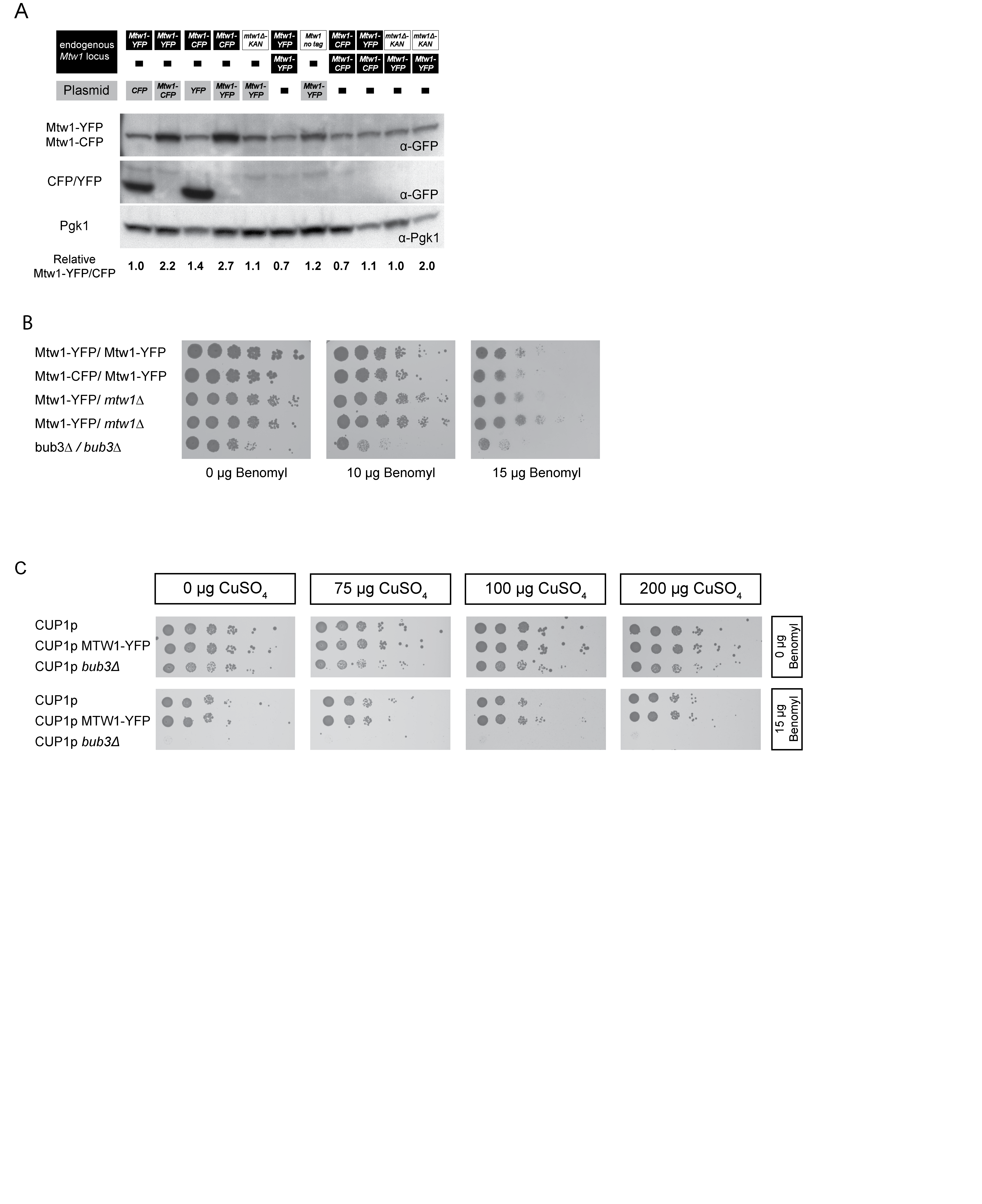

Supplement: S2 Fig — (A) Total Mtw1 protein increases in haploid and diploid strains from Fig 1. This is an expanded version of Fig 1F, showing a western blot of total cell extracts from both haploid and diploid cells. Quantification of cellular levels of Mtw1-YFP/-CFP relative to Pgk1 is shown below. (B) Diploid MTW1-YFP/mtw1Δ are haplo-sufficient. Serial dilutions of cells grown in YPD were spotted onto YPD plates containing benomyl diluted in DMSO. Cells were grown for 2 days at 30°C. (C) MTW1 over-expression does not affect growth or benomyl sensitivity. (TIF) [file pgen.1005855.s002.tif]

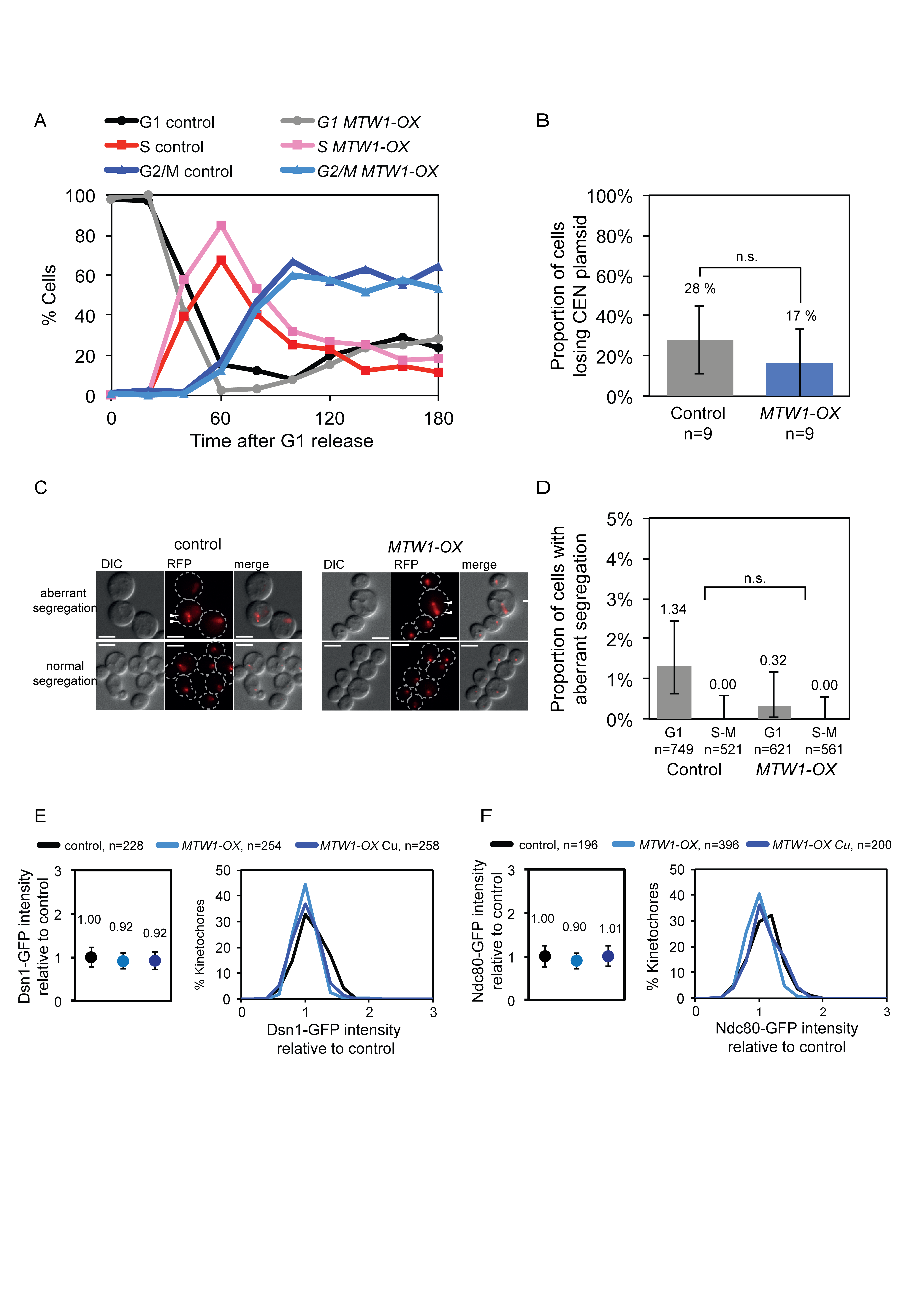

Supplement: S3 Fig — (A) Cell cycle progression is not altered in cells expressing ectopic MTW1. (B) The median proportion of cells losing a copy of a CEN plasmid after overnight growth without selection was not significantly different between cells containing an empty plasmid (control) and those containing MTW1 (MTW1-OX) (n = 9, error bars show standard deviation of the mean). (C) A tetracycline operator array, inserted at the URA3 locus of chromosome V, is marked with a tetracycline repressor linked to mRFP. Both normal and aberrant segregation of the chromosome V marker were seen in cells containing an empty plasmid (left panels) and MTW1 (right panels). Cell outlines are shown in the RFP image as dashed lines, arrowheads highlight aberrant segregation, the scale bar is 5μm. (D) The proportion of cells showing aberrant chromosome V segregation was not significantly different between cells containing an empty plasmid (control) and those containing MTW1 (MTW1-OX) (error bars show 95% binomial confidence intervals). (E-F) Quantitation of Dsn1-GFP (E) and Ndc80-GFP (F) kinetochore levels in control (black), low MTW1-OX (light blue) and high MTW1-OX (dark blue). Fluorescence intensity levels are normalised relative to control mean intensity. Left panel and right panels display the mean intensity ± standard deviation and the distribution of intensities, respectively. Ectopic MTW1 was expressed from a CUP1p. No additional copper was added to the low MTW1-OX cells. 100 μM CuSO4 was added to the high MTW1-OX cells for 3 hours before imaging. (TIF) [file pgen.1005855.s003.tif]

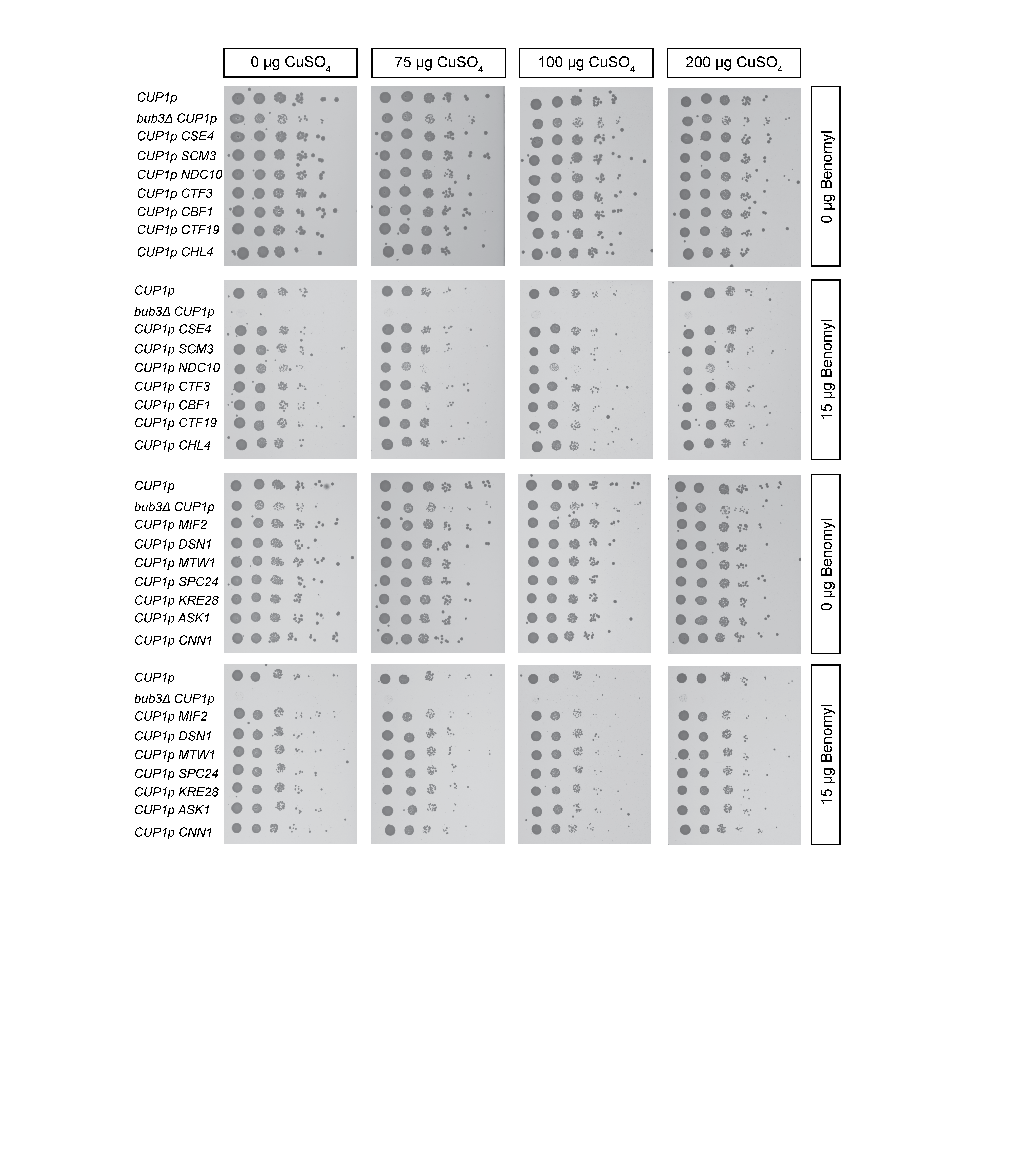

Supplement: S4 Fig — Serial dilutions of cells were spotted into synthetic media lacking leucine to select for plasmid, with several concentrations of benomyl and CuSO4. Cells spots were grown for 2 days at 30°C prior to imaging. (TIF) [file pgen.1005855.s004.tif]

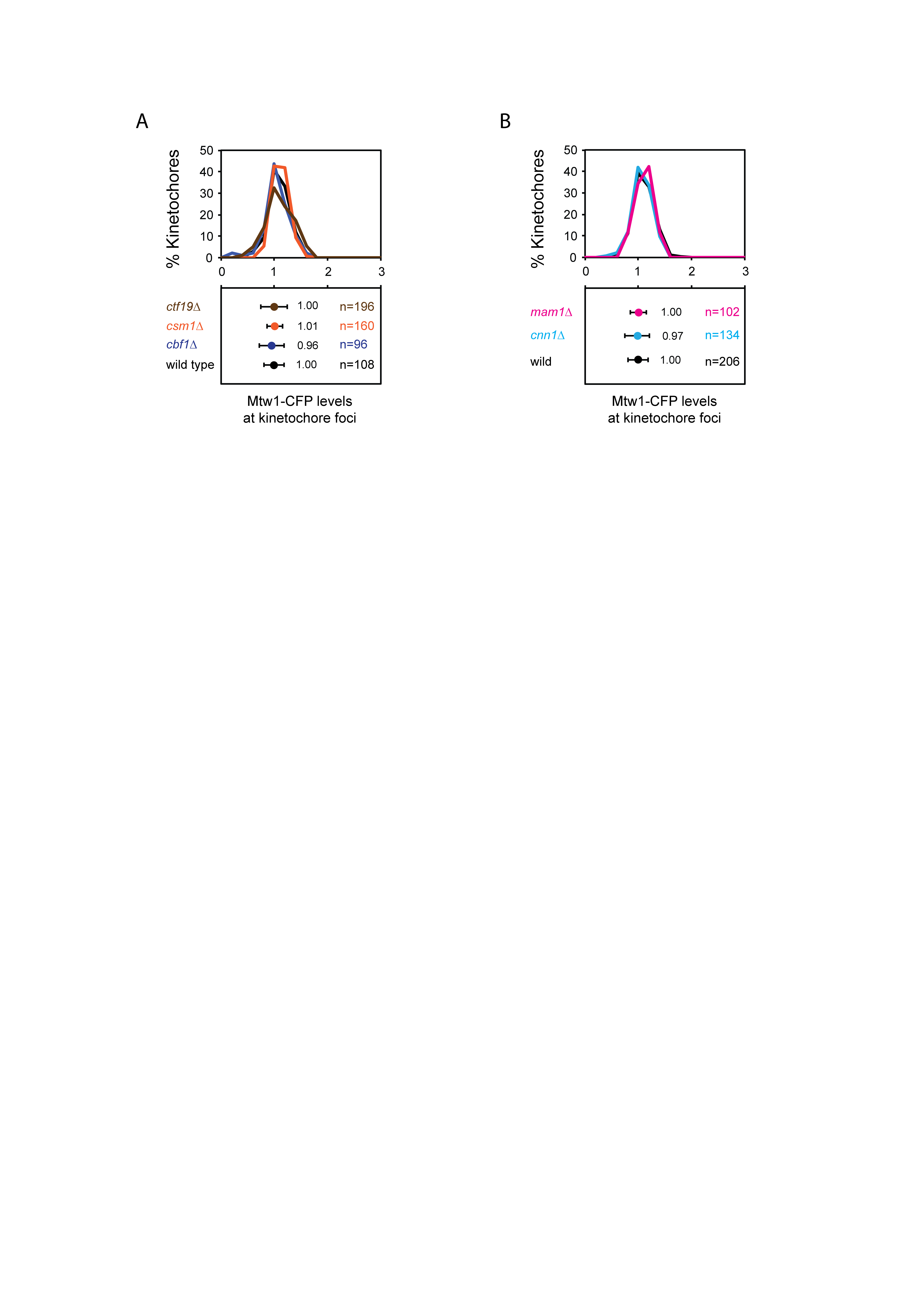

Supplement: S5 Fig — (A-B) Quantitation of Mtw1 kinetochore levels in ctf19Δ, csm1Δ, cbf1Δ, mam1Δ and cnn1Δ mutants. Fluorescence intensity levels are normalised relative to wild-type mean intensity. Top panels and bottom panels display the distribution of intensities and the mean intensity ± standard deviation, respectively. (TIF) [file pgen.1005855.s005.tif]

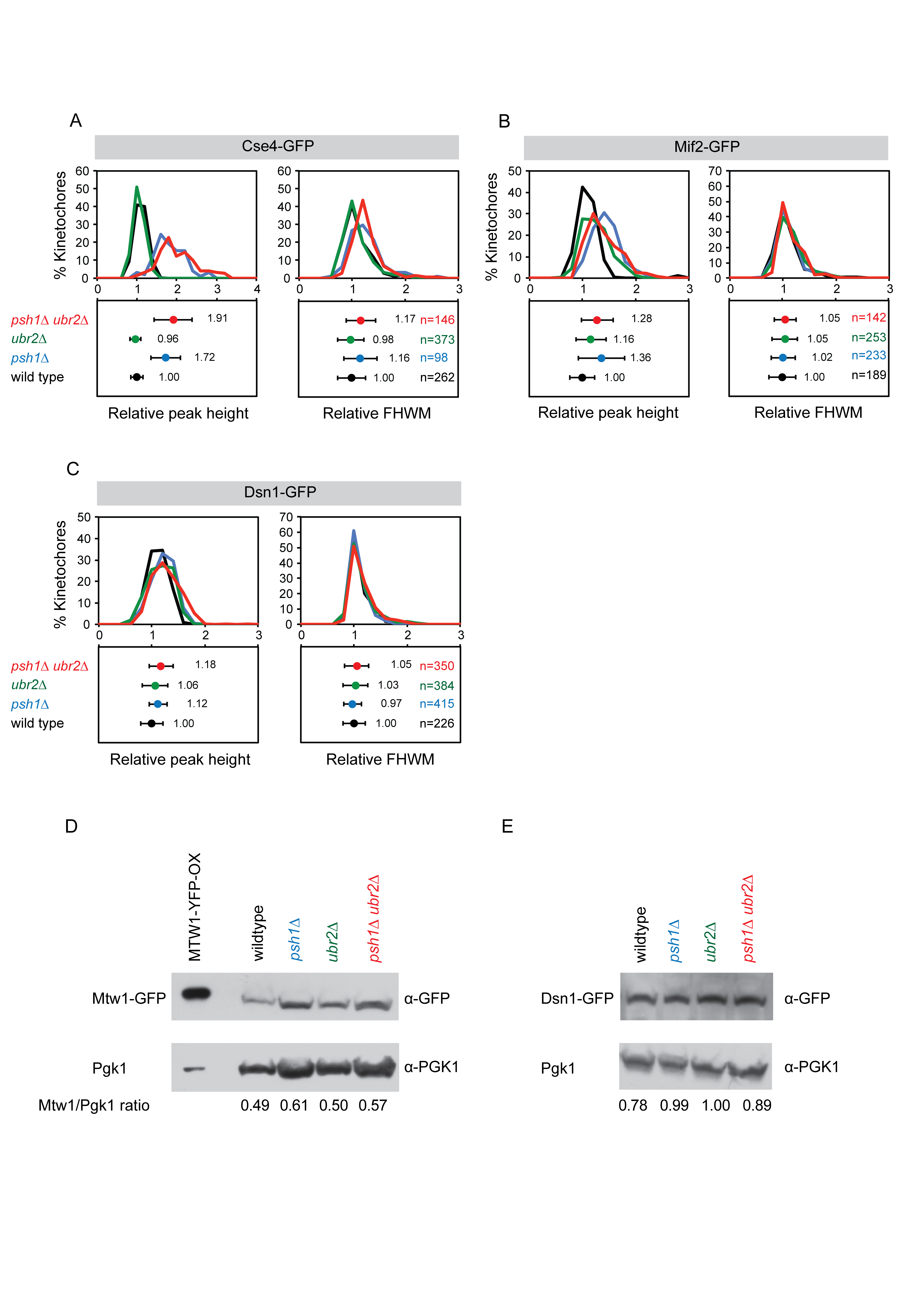

Supplement: S6 Fig — (A-C) Quantitation of size of kinetochore foci in wild type (black) psh1Δ (blue), ubr2Δ (green) and psh1Δ ubr2Δ (red) cells. Fluorescence peak height values and full width at half maximum (FHWM) values are normalised relative to wild-type mean intensity ± standard deviation. Top panels and bottom panels display the distribution of intensities and the mean intensity ± standard deviation, respectively. (A) Cse4-GFP. Peak height: wild type 1.00±0.15, psh1Δ 1.72±0.37***, ubr2Δ 0.96±0.14, psh1Δ ubr2Δ 1.91±0.48***. FHWM: wild type 1.00±0.26, psh1Δ 1.16±0.31***, ubr2Δ 0.98±0.25, psh1Δ ubr2Δ 1.17±0.28***. (B) Mif2-GFP. Peak height: wild type 1.00±0.24, psh1Δ 1.36±0.43***, ubr2Δ 1.16±0.28, psh1Δ ubr2Δ 1.28±0.30***. FHWM: wild type 1.00±0.25, psh1Δ 1.02±0.23, ubr2Δ 1.05±0.24, psh1Δ ubr2Δ 1.05±0.21. (C) Dsn1-GFP. Peak height: wild type 1.00±0.19, psh1Δ 1.12±0.21, ubr2Δ 1.06±0.23, psh1Δ ubr2Δ 1.18±0.28***. FHWM: wild type 1.00±0.21, psh1Δ 0.97±0.17, ubr2Δ 1.03±0.24, psh1Δ ubr2Δ 1.05±0.23 (D-E) Total Mtw1 and Dsn1 protein do not change in psh1Δ, ubr2Δ and psh1Δ ubr2Δ cells. Western blot of total cell extracts. Quantification of cellular levels of Mtw1-YFP/-CFP relative to Pgk1 is shown below. (TIF) [file pgen.1005855.s006.tif]

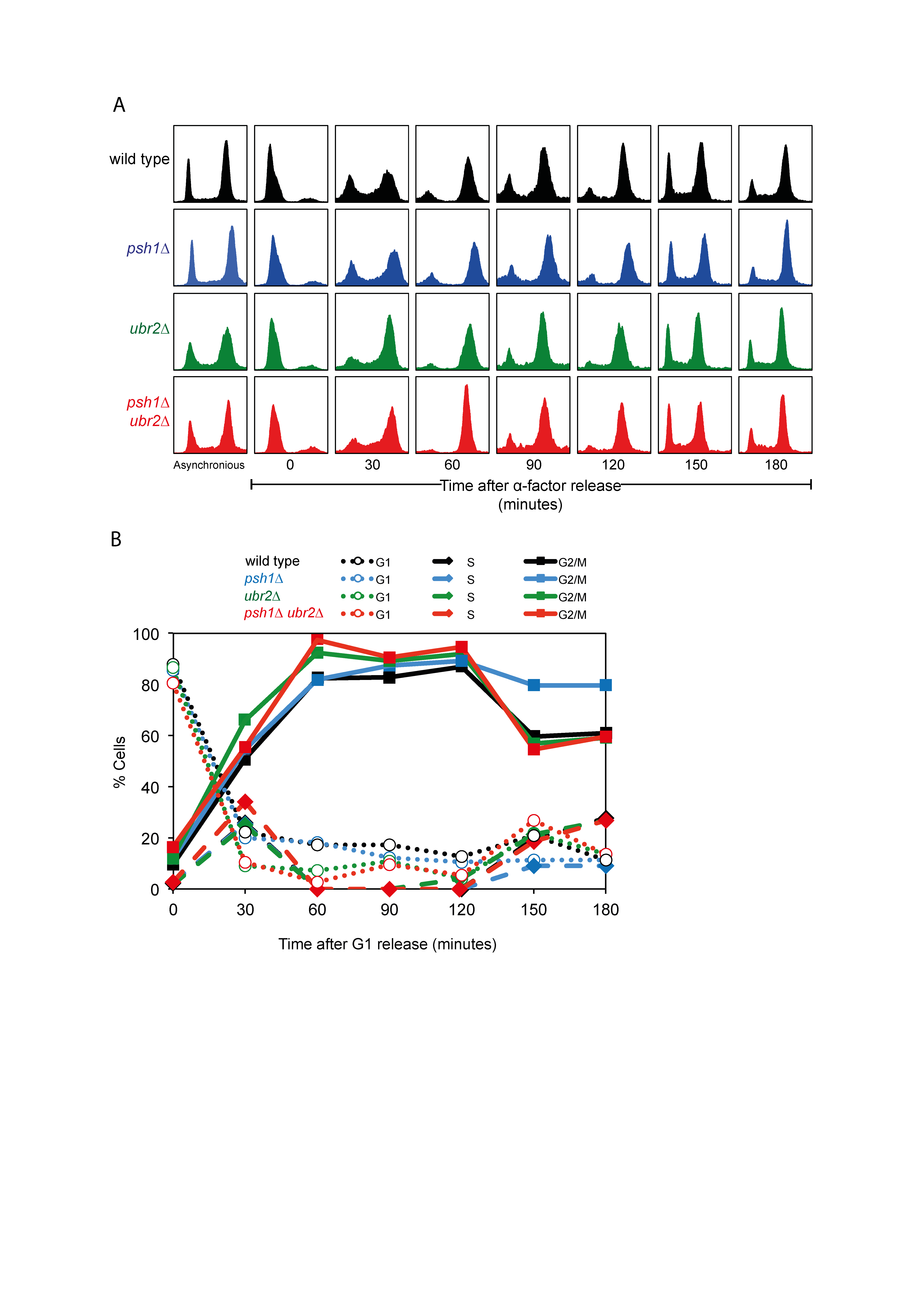

Supplement: S7 Fig — (A) Cell cycle profiles of wild type, phs1Δ, ubr2Δ, and psh1Δ ubr2Δ. MATa bar1Δ cells were synchronized in G1 with alpha-factor, and then released (Time 0). (B) Changes in the G1, S and G2/M populations during the course of the experiment. (TIF) [file pgen.1005855.s007.tif]

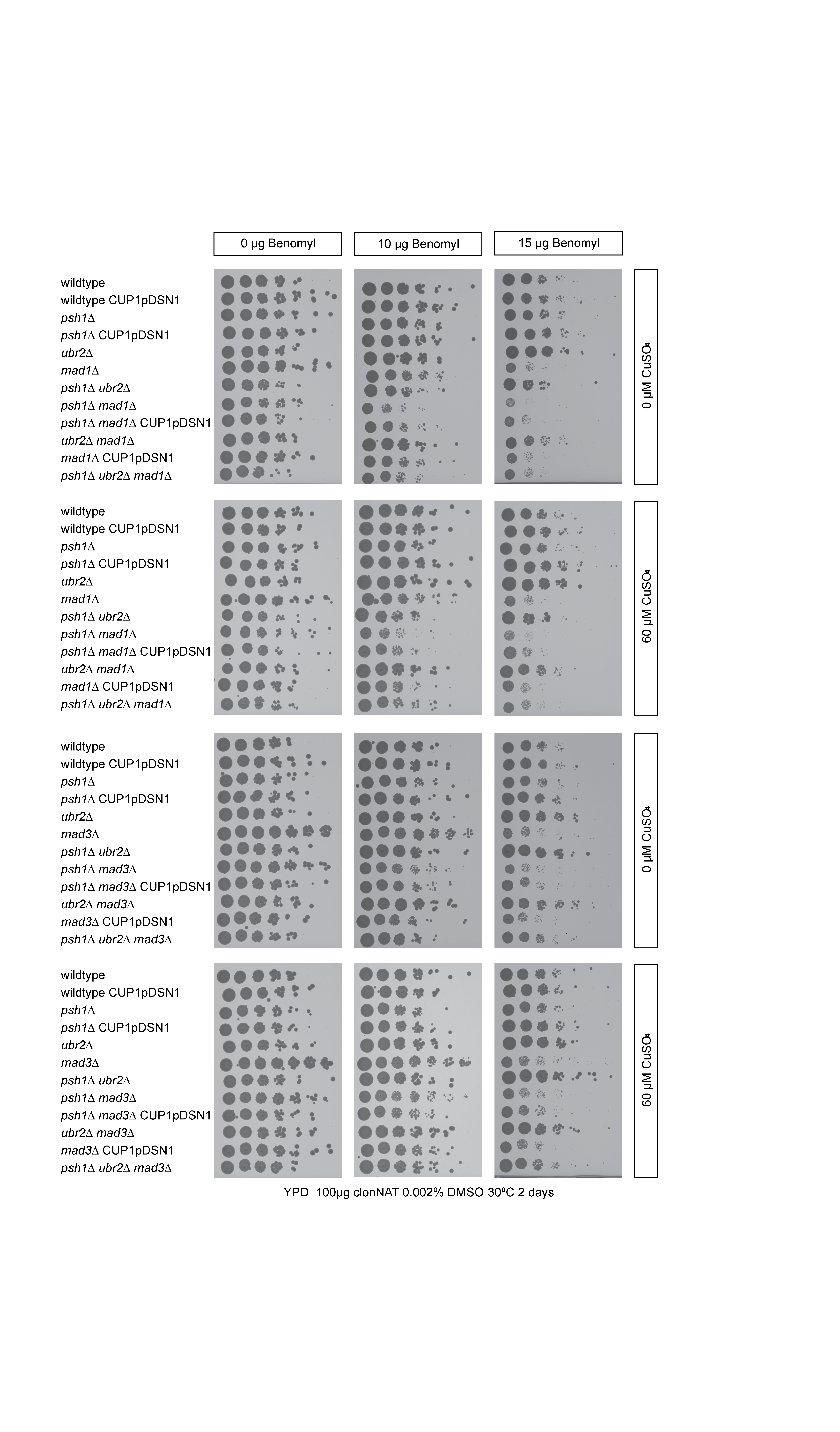

Supplement: S8 Fig — Serial dilutions of cells were spotted into YPD plates containing NAT to select for CUP1p DSN1-OX plasmid, with several concentrations of benomyl and CuSO4. Cells spots were grown for 2 days at 30°C prior to imaging. (TIF) [file pgen.1005855.s008.tif]
